# SLO BK Potassium Channels Couple Gap Junctions to Inhibition of Calcium Signaling in Olfactory Neuron Diversification

**DOI:** 10.1371/journal.pgen.1005654

**Published:** 2016-01-15

**Authors:** Amel Alqadah, Yi-Wen Hsieh, Jennifer A. Schumacher, Xiaohong Wang, Sean A. Merrill, Grethel Millington, Brittany Bayne, Erik M. Jorgensen, Chiou-Fen Chuang

**Affiliations:** 1 Department of Biological Sciences, University of Illinois at Chicago, Chicago, Illinois, United States of America; 2 Molecular and Developmental Biology Graduate Program, University of Cincinnati, Cincinnati, Ohio, United States of America; 3 Division of Developmental Biology, Cincinnati Children’s Hospital Research Foundation, Cincinnati, Ohio, United States of America; 4 Department of Biology and Howard Hughes Medical Institute, University of Utah, Salt Lake City, Utah, United States of America; New York University, UNITED STATES

## Abstract

The *C*. *elegans* AWC olfactory neuron pair communicates to specify asymmetric subtypes AWC^OFF^ and AWC^ON^ in a stochastic manner. Intercellular communication between AWC and other neurons in a transient NSY-5 gap junction network antagonizes voltage-activated calcium channels, UNC-2 (CaV2) and EGL-19 (CaV1), in the AWC^ON^ cell, but how calcium signaling is downregulated by NSY-5 is only partly understood. Here, we show that voltage- and calcium-activated SLO BK potassium channels mediate gap junction signaling to inhibit calcium pathways for asymmetric AWC differentiation. Activation of vertebrate SLO-1 channels causes transient membrane hyperpolarization, which makes it an important negative feedback system for calcium entry through voltage-activated calcium channels. Consistent with the physiological roles of SLO-1, our genetic results suggest that *slo-1* BK channels act downstream of NSY-5 gap junctions to inhibit calcium channel-mediated signaling in the specification of AWC^ON^. We also show for the first time that *slo-2* BK channels are important for AWC asymmetry and act redundantly with *slo-1* to inhibit calcium signaling. In addition, *nsy-5*-dependent asymmetric expression of *slo-1* and *slo-2* in the AWC^ON^ neuron is necessary and sufficient for AWC asymmetry. SLO-1 and SLO-2 localize close to UNC-2 and EGL-19 in AWC, suggesting a role of possible functional coupling between SLO BK channels and voltage-activated calcium channels in AWC asymmetry. Furthermore, *slo-1* and *slo-2* regulate the localization of synaptic markers, UNC-2 and RAB-3, in AWC neurons to control AWC asymmetry. We also identify the requirement of *bkip-1*, which encodes a previously identified auxiliary subunit of SLO-1, for *slo-1* and *slo-2* function in AWC asymmetry. Together, these results provide an unprecedented molecular link between gap junctions and calcium pathways for terminal differentiation of olfactory neurons.

## Author Summary

Cell type diversity is important for the nervous system to function properly. Asymmetric differentiation of neurons along the left-right axis is one way to achieve diversity; however, the molecular mechanisms used to establish neuronal asymmetry are only partly understood. In the nematode *C*. *elegans*, the AWC sensory neuron pair displays stochastic asymmetric identities. Communication between neurons, including the two AWC neurons, through gap junctions inhibits calcium channels in one AWC neuron, resulting in two distinct AWC identities. How gap junctions repress calcium channels in one AWC is not well understood. We show that voltage- and calcium-activated potassium channels provide a molecular link between gap junctions and calcium channels to establish AWC neuronal asymmetry. We show that potassium channels are asymmetrically expressed in AWC neurons, which is dependent on gap junctions. We also find that potassium channels localize close to calcium channels in AWC, suggesting they may functionally couple to establish AWC asymmetry. In addition, our results show that potassium channels regulate the localization of synaptic markers in AWC for asymmetry. Furthermore, we identify an auxiliary subunit of the potassium channels required for their function in establishing AWC asymmetry. These results shed light on mechanisms used to diversify neuronal cell types.

## Introduction

The nervous system generates a tremendous diversity of cell types that enable formation of functional neural circuits for information processing and mediating behaviors. Cellular diversity is especially important in the developing sensory system as it allows animals to detect different cues in the environment. However, the molecular mechanisms that generate neuronal diversification are only partly understood. One way to generate cellular diversity in the nervous system is to specify different fates and functions of individual cell types across the left-right axis. Left-right asymmetry in the nervous system is present throughout the animal kingdom [1–3]. For example, anatomical and functional asymmetries in the human nervous system have been described, such as the greater size of the planum temporale in the left hemisphere, and the localization of language to the left hemisphere of the brain [4]. Defects in brain asymmetry have been correlated with various neurological diseases such as dyslexia and schizophrenia [5]. In the *C*. *elegans* nervous system, two pairs of head sensory neurons display molecular and functional asymmetries: the ASE taste neurons and the AWC olfactory neurons [6–9].

The left and right AWC olfactory neurons appear symmetric at the anatomical and morphological level. However, the two AWC neurons differentiate asymmetrically into two distinct subtypes, one default AWC^OFF^ and one induced AWC^ON^, at both molecular and functional levels in late embryogenesis [10–12]. The AWC^ON^ subtype expresses the G-protein coupled receptor (GPCR) gene *str-2* and functions to detect the odorant butanone [11,12]. The AWC^OFF^ subtype expresses the GPCR gene *srsx-3* and functions to sense the odorant 2,3-pentanedione [12,13]. AWC asymmetry is stochastic, such that the AWC^ON^ subtype is induced on the left side of the animal in 50% of the population and on the right side of the animal in the other 50% [11]. AWC asymmetry is maintained throughout the life of an animal [11,14,15].

The default AWC^OFF^ subtype is specified by a calcium-activated protein kinase pathway. In this pathway, calcium entry through voltage-gated calcium channels (the pore-forming α1 subunits UNC-2/N-type or EGL-19/L-type and the regulatory α2δ subunit UNC-36) activates a kinase cascade that consists of UNC-43 calcium/calmodulin dependent protein kinase (CaMKII), the TIR-1 (Sarm1) adaptor protein, NSY-1 MAP kinase kinase kinase (MAPKKK), and SEK-1 MAPKK [10,11,16,17]. TIR-1 assembles a calcium-signaling complex containing UNC-43 (CaMKII) and NSY-1 (MAPKKK) at postsynaptic sites in the AWC axons, in a manner dependent on microtubules and the kinesin motor protein UNC-104, to promote the AWC^OFF^ subtype [10,18]. Intercellular calcium signaling through a transient embryonic neural network, formed between AWC and other neurons via the NSY-5 gap junction protein innexin, coordinates precise AWC asymmetry [19]. In addition, NSY-5 and the NSY-4 claudin-like protein function in parallel to antagonize calcium signaling through *mir-71*-mediated downregulation of *tir-1* expression in the AWC^ON^ subtype [20–22]. However, the mechanism by which NSY-5 gap junctions and NSY-4 claudin suppress *unc-2/unc-36* and *egl-19/unc-36* calcium signaling to induce the AWC^ON^ subtype is only beginning to be understood.

The *ky389* and *ky399* alleles were identified from a forward genetic screen for mutants with two AWC^ON^ neurons (2AWC^ON^ phenotype) [11]. The *ky389* and *ky399* mutations were revealed as gain-of-function (gf) alleles of *slo-1* in a study demonstrating a central role of *slo-1* in behavioral response to ethanol [23]. *slo-1* encodes a conserved voltage- and calcium-activated large conductance BK potassium channel [24,25]. Activation of SLO-1 (Slo1) channels causes hyperpolarization of the cell membrane, thereby reducing cellular excitability and limiting calcium entry through voltage-gated calcium channels [26]. The 2AWC^ON^ phenotype of *slo-1(gf)* mutants suggests a sufficient role of *slo-1(gf)* in promoting AWC^ON^. However, the effect of *slo-1* loss-of-function mutations on AWC asymmetry and the mechanism by which *slo-1* functions to control AWC asymmetry remained unaddressed. Here we demonstrate that both *slo-1* and *slo-2* BK channels are necessary for the establishment of AWC asymmetry. We show that *slo-1* and *slo-2* act redundantly downstream of *nsy-5* (innexin gap junction protein) and in parallel with *nsy-4* (claudin) to antagonize the function of *unc-2* and *egl-19* (voltage-gated calcium channels) in the induced AWC^ON^ subtype. Asymmetric expression of *slo-1* and *slo-2* in the AWC^ON^ neuron, which is dependent on NSY-5 and NSY-4, is necessary and sufficient for AWC asymmetry. In addition, SLO-1 and SLO-2 BK channels localize close to UNC-2 and EGL-19 voltage-gated calcium channels, suggesting that SLO channels may inhibit calcium channels through functional coupling and negative feedback. Our results also suggest that *slo-1* and *slo-2* may regulate AWC communication to control AWC asymmetry through modulating UNC-2 synaptic puncta and synaptic vesicle clustering. Thus, our study identifies an unprecedented role of SLO BK potassium channels in mediating transient gap junction signaling for inhibition of a calcium channel-activated kinase cascade in terminal differentiation of olfactory neurons.

## Results

### *slo-1* and *slo-2* BK potassium channels act redundantly to establish AWC asymmetry

**Fig 1:**
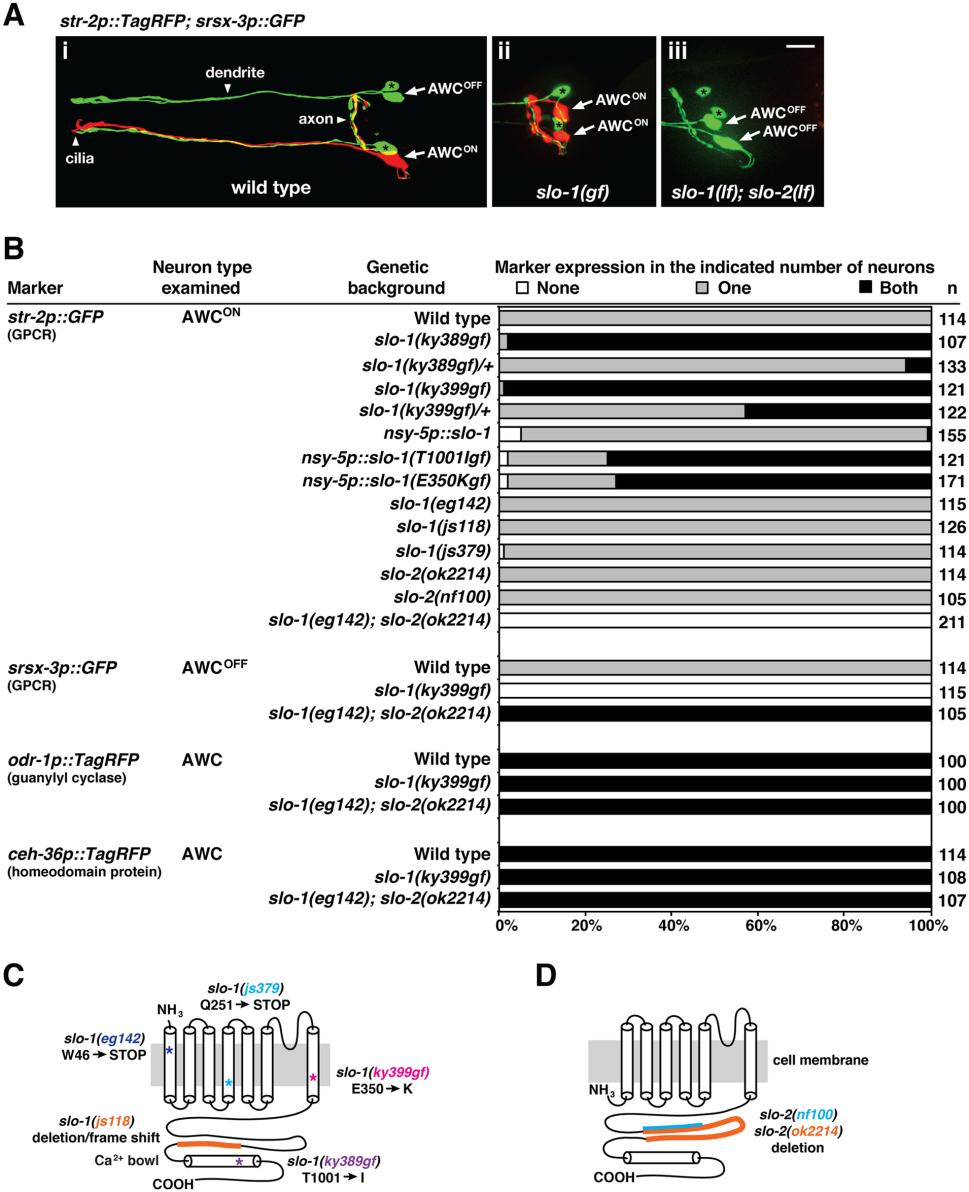
*slo-1* and *slo-2* act redundantly to promote the AWC^ON^ neuronal subtype. (**A**) Images of wild type (i), *slo-1(ky399gf)* mutants (ii), and *slo-1(eg142lf); slo-2(ok2214lf)* double mutants (iii) expressing the transgene *str-2p*::*TagRFP* (AWC^ON^ marker); *srsx-3p*::*GFP* (AWC^OFF^ marker) in the adult stage. (i) Wild-type animals have one AWC^ON^ neuron and one AWC^OFF^ neuron. (ii) *slo-1(ky399gf)* mutants express *str-2p*::*TagRFP* in both AWC neurons, showing a 2AWC^ON^ phenotype. (iii) *slo-1(eg142lf); slo-2(ok2214lf)* double mutants express *srsx-3p*::*GFP* in both AWC cells, displaying a 2AWC^OFF^ phenotype. *srsx-3p*::*GFP* is also expressed in two AWB cells, in addition to the AWC^OFF^ cell. Arrows indicate the AWC cell body; asterisks indicate the AWB cell body. Scale bar, 10 μm. Anterior is at left and ventral is at bottom. (**B**) Expression of AWC markers in *slo-1* and *slo-2* mutants. (**C, D**) Schematic representation of SLO-1 (C) and SLO-2 (D) proteins with mutation sites and resulting residue changes indicated for the mutants used. Asterisks indicate missense or nonsense mutations. Orange and blue lines indicate regions deleted in *slo-1(lf)* and *slo-2(lf)* mutants.

Wild-type animals have one default AWC^OFF^ neuron, expressing the GPCR gene *srsx-3*, and one induced AWC^ON^ neuron, expressing the GPCR gene *str-2* (Fig 1Ai and 1B). Both *slo-1(ky389gf)* and *slo-1(ky399gf)* mutations resulted in expression of the AWC^ON^ marker *str-2* in two AWC neurons (2AWC^ON^ phenotype) (Fig 1Aii, 1B and 1C), as shown previously [11]. Both *slo-1(ky389gf)/+* and *slo-1(ky399gf)/+* heterozygous animals displayed a less penetrant 2AWC^ON^ phenotype (Fig 1B), confirming their characterization as dominant gain-of-function mutants. We set out to further characterize the AWC phenotypes of *slo-1(gf)* mutants. We found that the AWC^OFF^ marker *srsx-3* was not expressed in either of the AWC neurons of *slo-1(gf)* mutants (Fig 1Aii and 1B), consistent with the 2AWC^ON^ phenotype of the mutants (Fig 1Aii and 1B). In addition, overexpression of *slo-1(T1001Igf)* and *slo-1(E350Kgf)*, containing *ky389gf* and *ky399gf* mutations, respectively, in AWC in a wild-type background also caused a strong 2AWC^ON^ phenotype (73%-75%) (Fig 1B). However, overexpression of wild-type *slo-1* only caused a very weak 2AWC^ON^ phenotype (1%) when injected at the same concentration as the *slo-1(T1001Igf)* and *slo-1(E350Kgf)* transgenes (Fig 1B). Our results are consistent with the previous electrophysiological study suggesting that *slo-1(ky389gf)* and *slo-1(ky399gf)* mutations result in increased SLO-1 channel activity in dopaminergic neurons [23].

Although *slo-1(gf)* mutants caused a strong 2AWC^ON^ phenotype, we found that loss-of-function (lf) mutations in *slo-1* did not display any defects in AWC asymmetry (Fig 1B and 1C). This suggests that *slo-1* may function redundantly with other genes to establish AWC asymmetry. Since *slo-2* encodes the only other calcium-activated SLO-like potassium channel in *C*. *elegans* and its expression overlaps with *slo-1* [24,27,28], we hypothesized that *slo-1* and *slo-2* may function redundantly to control AWC asymmetry. Similar to *slo-1(lf)* mutants, *slo-2(lf)* mutants did not exhibit abnormalities in AWC asymmetry (Fig 1B and 1D). However, *slo-1(eg142lf); slo-2(ok2214lf)* double mutants had a complete penetrance of two AWC^OFF^ neurons (2AWC^OFF^ phenotype): the expression of the AWC^ON^ marker *str-2* was lost and the AWC^OFF^ marker *srsx-3* was expressed in both AWC neurons (Fig 1Aiii and 1B). Together, these results suggest that *slo-1* and *slo-2* have essential and redundant roles in promoting the AWC^ON^ subtype.

To determine whether *slo-1* and *slo-2* affect general AWC fate, we examined the expression of two general AWC markers, the guanylyl cyclase gene *odr-1* and the homeodomain protein encoding gene *ceh-36*, both of which are expressed in both AWC neurons in wild-type animals. Both *slo-1(ky399gf)* and *slo-1(eg142lf); slo-2(ok2214lf)* double mutants displayed normal expression of *odr-1* and *ceh-36* (Fig 1B), suggesting that general AWC identity is not affected by the mutations.

### *slo-1* and *slo-2* act downstream of *nsy-5* to antagonize calcium channel-mediated signaling in promoting the AWC^ON^ subtype

**Fig 2:**
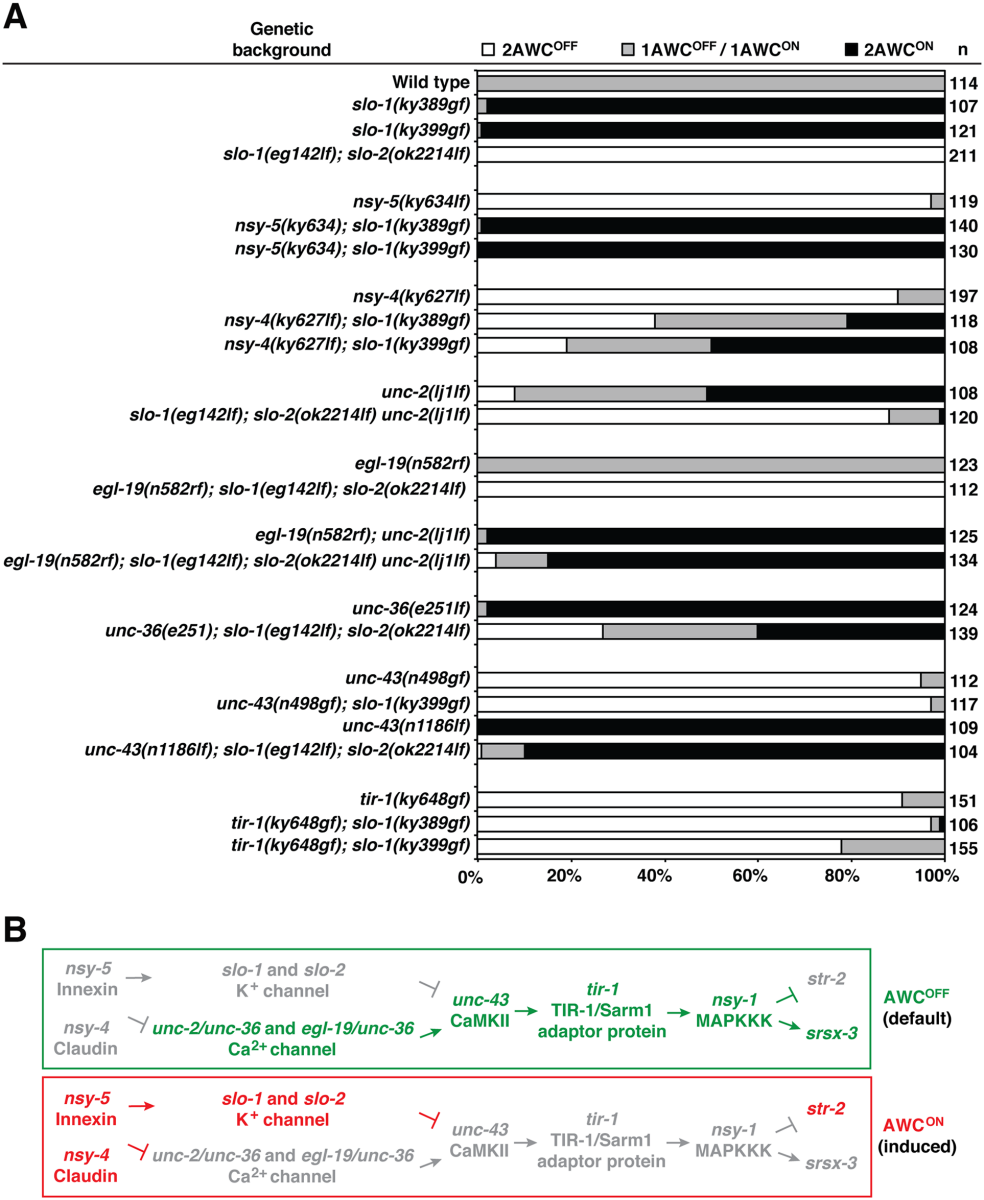
*slo-1* and *slo-2* act downstream of *nsy-5* to antagonize the function of voltage-gated calcium channel-activated kinase cascade in promoting AWC^ON^. (**A**) Double and triple mutant analysis of *slo-1(ky389gf)*, *slo-1(ky399gf)*, and *slo-1(eg142lf); slo-1(ok2214lf)* animals with mutants of known genes involved in establishment of AWC asymmetry. 2AWC^ON^, both AWC cells express *str-2*; 1AWC^OFF^/AWC^ON^, only one of the two AWC cells expresses *str-2*; 2AWC^OFF^, neither AWC cell expresses *str-2*. (**B**) The genetic pathway that demonstrates possible relationships between *slo-1*, *slo-2* and other genes required for AWC asymmetry. Genes in green represent AWC^OFF^ promoting, genes in red represent AWC^ON^ promoting, and those in grey represent less active or inactive genes.

The 2AWC^ON^ phenotype of *slo-1(gf)* mutants and the 2AWC^OFF^ phenotype of *slo-1(lf); slo-2(lf)* double mutants (Figs 1Aii, 1Aiii, 1B and 2A) indicate that the two BK potassium channels function to promote the induced AWC^ON^ subtype. To shed light on how *slo-1* and *slo-2* promote AWC^ON^, we investigated where they are located within the AWC asymmetry pathway by generating double and triple mutants of *slo-1*, *slo-2*, and other genes previously implicated in AWC asymmetry (Fig 2A).

The *slo-1(gf)* 2AWC^ON^ mutants were crossed with 2AWC^OFF^ mutants including *nsy-5/*innexin*(lf)*, *nsy-4/*claudin*(lf)*, *unc-43/*CaMKII*(gf)*, and *tir-1*/Sarm1*(gf)* [11,18,20,21]. The 2AWC^OFF^ phenotype of *nsy-5(lf)* mutants was completely suppressed by *slo-1(gf)* mutants (Fig 2A), suggesting that *slo-1* acts downstream of *nsy-5* gap junctions to specify AWC^ON^ (Fig 2B). *nsy-4(lf); slo-1(gf)* double mutants had mixed 2AWC^ON^ and 2AWC^OFF^ phenotypes (Fig 2A), suggesting that *slo-1* acts in parallel with *nsy-4* claudin in promoting AWC^ON^ (Fig 2B). Furthermore, the 2AWC^ON^ phenotype of *slo-1(gf)* was nearly completely suppressed by *unc-43(gf)* and *tir-1(gf)* mutations (Fig 2A); the 2AWC^OFF^ phenotype of *slo-1(lf); slo-2(lf)* double mutants was almost completely suppressed by the *unc-43(lf)* mutants (Fig 2A), which is consistent with the previous notion that *slo-1* acts upstream of *unc-43* (CaMKII) [16] (Fig 2B).

Since our genetic results put *slo-1* and *slo-2* (BK potassium channels) at a position similar to *unc-2/unc-36* and *egl-19/unc-36* (voltage-gated calcium channels) in the AWC asymmetry pathway, we examined the genetic interaction of *slo-1* and *slo-2* with *unc-36*. *unc-36(e251lf)* mutants have a strong 2AWC^ON^ phenotype [11](Fig 2A) and were crossed into the *slo-1(lf); slo-2(lf)* 2AWC^OFF^ mutants. We found that the 2AWC^ON^ phenotype of *unc-36(lf)* and the 2AWC^OFF^ phenotype of *slo-1(lf); slo-2(lf)* were significantly mutually suppressed in *unc-36(lf); slo-1(lf); slo-2(lf)* triple mutants (Fig 2A). These genetic analyses suggest antagonistic and parallel functions of BK potassium channels (*slo-1* and *slo-2*) and voltage-gated calcium channels (*unc-36*) in AWC asymmetry (Fig 2B). *unc-2* and *egl-19*, both of which encode α1 subunits of voltage-activated calcium channels, were shown to have partially redundant functions in AWC asymmetry [13]. *unc-2(lj1lf)* mutants had a mixed 2AWC^ON^ and 2 AWC^OFF^ phenotype, while a reduction of function (rf) allele of *egl-19* did not display any AWC asymmetry defects. However, *egl-19(rf); unc-2(lf)* double mutants caused a strong 2AWC^ON^ phenotype reminiscent of *unc-36(lf)* mutants [13] (Fig 2A), which supports partially redundant functions of *egl-19* and *unc-2* in AWC asymmetry. To test whether *unc-2* and *egl-19* interact with *slo-1* and *slo-2* to establish AWC asymmetry, we determined genetic relationship between *unc-2*, *egl-19*, *slo-1*, and *slo-2*. The 2AWC^OFF^ phenotype of *slo-1(lf); slo-2(lf)* was only slightly suppressed or not suppressed in *slo-1(lf); slo-2(lf) unc-2(lf)* or *egl-19(rf)*; *slo-1(lf); slo-2(lf)* mutants, respectively (Fig 2A). These results suggest that *unc-2(lf)* or *egl-19(rf)* alone are not sufficient to suppress the *slo-1(lf); slo-2(lf)* 2AWC^OFF^ phenotype. However, *egl-19(rf); slo-1(lf); slo-2(lf) unc-2(lf)* quadruple mutants displayed a high 2AWC^ON^ phenotype, which resembled *egl-19(rf); unc-2(lf)* mutants (Fig 2A). This result suggests that *unc-2* and *egl-19* act redundantly to antagonize *slo-1* and *slo-2* function to promote the AWC^OFF^ identity.

Taken together, these genetic results suggest that *slo-1* and *slo-2* (BK potassium channels) act downstream of *nsy-5* (innexin) and in parallel with *nsy-4* (claudin) to antagonize the function of *unc-2/unc-36* and *egl-19/unc-36* (voltage-gated calcium channels) to induce AWC^ON^ (Fig 2B).

### *slo-1* and *slo-2* are expressed asymmetrically in the AWC^ON^ neuron

**Fig 3:**
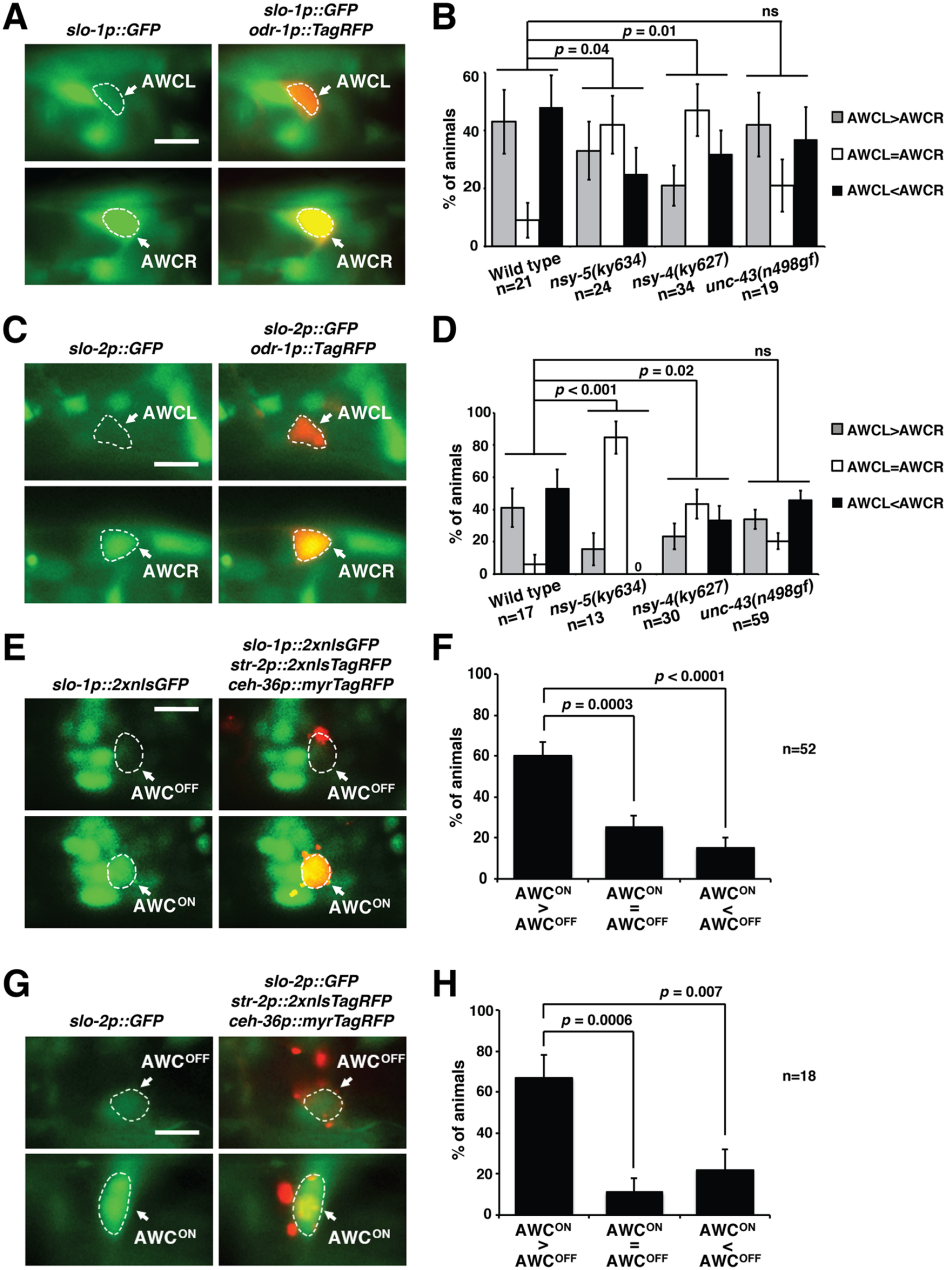
*slo-1* and *slo-2* are expressed asymmetrically in the AWC^ON^ neuron. (**A, C**) Images of wild-type L1 animals showing expression of *slo-1p*::*GFP* (A) and *slo-2p*::*GFP* (C) at a higher level in AWCR (bottom panels) than in AWCL (top panels). Both AWCL and AWCR were labeled by *odr-1p*::*TagRFP*. The cell body of both AWC cells is outlined by dashed lines. Scale bar, 5 μm. Anterior is at left and ventral is at bottom. (**B, D**) Quantification of asymmetric expression of *slo-1p*::*GFP* (B) and *slo-2p*::*GFP* (D) in AWCL and AWCR in wild type and mutants defective in AWC asymmetry. The single focal plane with the brightest fluorescence in each AWC was selected from the acquired image stack and compared for fluorescence intensity. The fluorescence intensity of *slo-1p*::*GFP* and *slo-2p*::*GFP* was compared using visual quantitative scoring between AWCL and AWCR in each animal, as previously performed [7,22,58]. If no obvious difference in fluorescence intensity between the two AWC cells was observed, the animal was categorized as AWCL = AWCR. If an obvious difference in fluorescence intensity was observed between AWCL and AWCR, the animal was assigned to AWCL > AWCR or AWCR > AWCL. For both *slo-1p*::*GFP* and *slo-2p*::*GFP*, the visual quantification of fluorescence was performed by the same individual. Only animals with visible expression in both AWC neurons were used in the analysis. *p* values were calculated using Fisher’s exact test. ns, not significant. Error bars indicate standard error of proportion. (**E, G**) Representative images of wild-type L1 animals expressing *slo-1p*::*2xnlsGFP* (E) and *slo-2p*::*GFP* (G) in AWC^ON^ (bottom panel) but not in AWC^OFF^ (top panel). Both AWC neurons were marked with *ceh-36p*::*myrTagRFP*. AWC^ON^ cells were marked by *str-2p*::*2xnlsTagRFP*, and AWC^OFF^ neurons were defined by lack of *str-2p*::*2xnlsTagRFP*. Scale bar, 5 μm. Anterior is at left and ventral is at bottom. (**F, H**) Quantification of *slo-1p*::*2xnlsGFP* (F) and *slo-2p*::*GFP* (H) expression in AWC^ON^ and AWC^OFF^. The single focal plane with the brightest fluorescence in each AWC was selected from the acquired image stack and compared for fluorescence intensity. The fluorescence intensity of *slo-1p*::*2xnlsGFP* and *slo-2p*::*GFP* was compared using visual quantitative scoring between AWC^ON^ and AWC^OFF^ in each animal, as previously performed [7,22,58]. Each animal was categorized into one of three categories: AWC^ON^ = AWC^OFF^, AWC^ON^ > AWC^OFF^, and AWC^OFF^ > AWC^ON^ based on the comparison of GFP intensities between AWC^ON^ and AWC^OFF^ cells of the same animal. *p* values were calculated using a *Z*-test. Error bars indicate standard error of proportion.

Both *slo-1* and *slo-2* are widely expressed in neurons and muscles [25,28]. To determine if *slo-1* and *slo-2* are expressed in AWC neurons, we crossed *odr-1p*::*TagRFP* (expressed in both AWC neurons) with *slo-1p*::*GFP* and *slo-2p*::*GFP* transgenic strains. We found that GFP expressed from *slo-1p* and *slo-2p* was colocalized with the AWC marker *odr-1p*::*TagRFP* (Fig 3A and 3C), suggesting that *slo-1* and *slo-2* are expressed in AWC.

To determine if *slo-1* and *slo-2* are expressed asymmetrically in AWC neurons, we compared their respective expression level in AWC left (AWCL) and AWC right (AWCR). Although *slo-1* and *slo-2* are expressed in both AWC neurons in the majority of wild-type animals, both *slo-1* and *slo-2* are asymmetrically expressed in AWCL or AWCR in a stochastic manner (Fig 3B and 3D, AWCL>AWCR versus AWCL<AWCR are indistinguishable). Random asymmetry of *slo-1* and *slo-*2 expression in AWC neurons is consistent with the stochastic nature of AWC asymmetry. In contrast, *nsy-5(ky634lf)* and *nsy-4(ky627lf)* mutants exhibited a significant increase in the percentage of animals that expressed *slo-1* and *slo-2* symmetrically in AWCL and AWCR (Fig 3B and 3D, AWCL = AWCR). This data suggests that *nsy-5* (innexin) and *nsy-4* (claudin) are required for the asymmetric expression of *slo-1* and *slo-2* in AWC neurons, and is consistent with our genetic analysis demonstrating that *nsy-5* acts in parallel with *nsy-4* to promote AWC^ON^ through *slo-1* and *slo-2* (Fig 2B). As a control, we examined the asymmetric expression of *slo-1* and *slo-2* in *unc-43(n498gf)/*CaMKII mutants, which cause a 2AWC^OFF^ phenotype, similar to that caused by *nsy-5(ky634lf)* and *nsy-4(ky627lf)*. We found that the asymmetric expression of both *slo-1* and *slo-2* was unaffected by the *unc-43(n498gf)* mutation (Fig 3B and 3D). This suggests that *unc-43* (CaMKII) does not regulate the expression of *slo-1* and *slo-2*, and is consistent with our genetic results, which place *slo-1* and *slo-2* upstream of *unc-43/*CaMKII. The result also supports that the effect of *nsy-4* and *nsy-5* loss-of-function mutations on asymmetric expression of *slo-1* and *slo-2* was not due to the 2AWC^OFF^ phenotype.

We also compared expression level of *slo-1* and *slo-2* in AWC^ON^ and AWC^OFF^, and found that *slo-1* and *slo-2* are expressed predominantly in the AWC^ON^ cell (Fig 3E–3H). These results are consistent with the hypothesis that *slo-1* and *slo-2* promote AWC^ON^ in a cell-autonomous manner.

### *slo-1* and *slo-2* act cell autonomously to specify AWC^ON^

**Fig 4:**
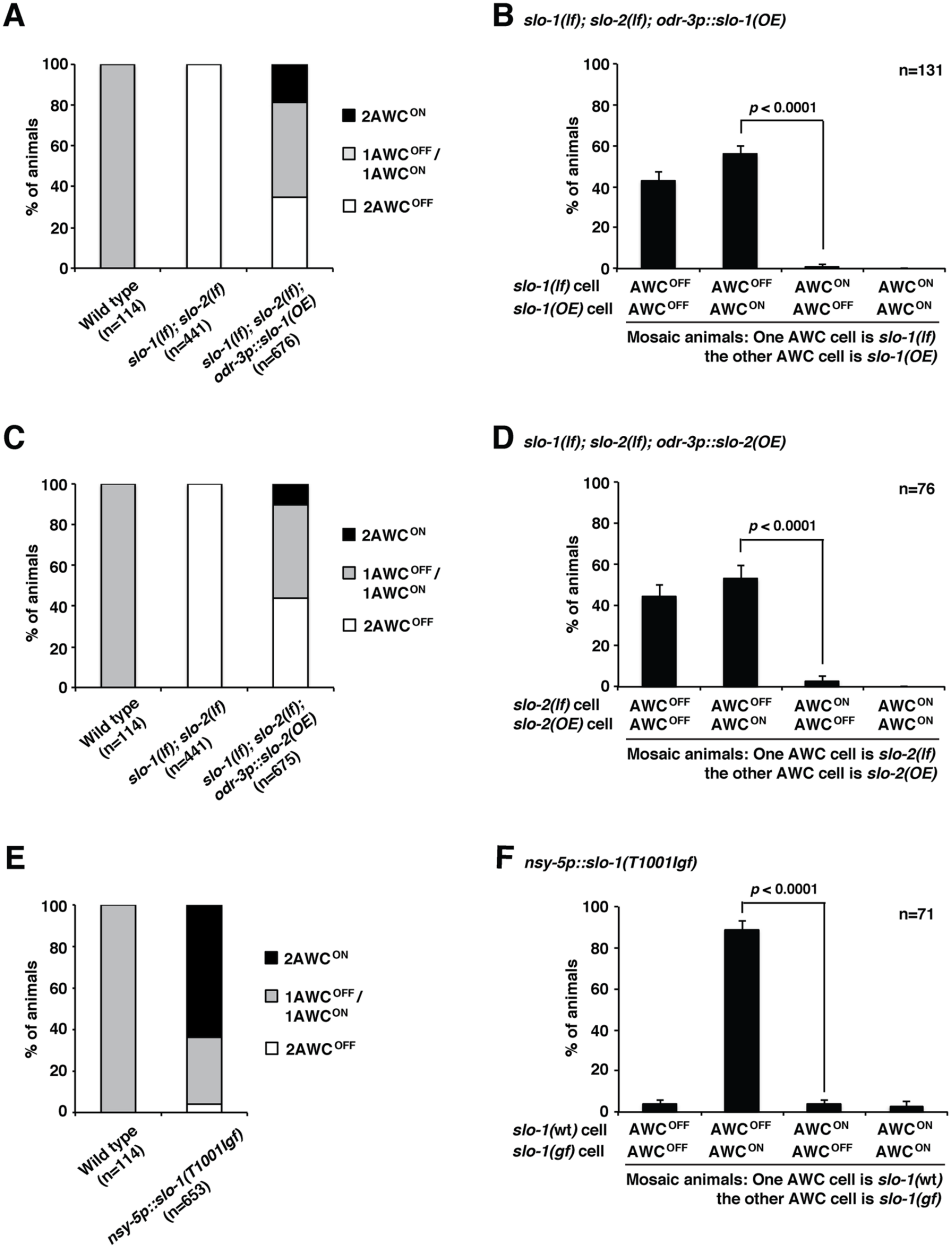
*slo-1* and *slo-2* act cell autonomously in promoting the AWC^ON^ fate. (**A**, **C**) AWC phenotypes in wild type, *slo-1(eg142lf); slo-2(ok2214lf)*, and *slo-1(eg142lf); slo-2(ok2214lf)* expressing extrachromosomal transgenes *odr-3p*::*slo-1(OE); odr-1p*::*DsRed* (**A**) or *odr-3p*::*slo-2(OE); odr-1p*::*DsRed* (**C**). (**B**) AWC phenotypes of *slo-1(eg142lf); slo-2(ok2214lf)* mosaic animals containing the extrachromosomal transgene *odr-3p*::*slo-1(OE)* in only one AWC cell, inferred by the presence of the coinjection marker *odr-1p*::*DsRed* (normally expressed in both AWC). The data was derived from a subset of data in (**A**). (**D**) AWC phenotypes of *slo-1(eg142lf); slo-2(ok2214lf)* mosaic animals containing the extrachromosomal transgene *odr-3p*::*slo-2(OE); odr-1p*::*DsRed* in only one AWC cell. The data was derived from a subset of data in (**C**). (**E**) AWC phenotypes in wild-type animals expressing the extrachromosomal transgene *nsy-5p*::*slo-1(T1001Igf); odr-1p*::*DsRed*. *slo-1(T1001Igf)* contains the *ky389gf* mutation. (**F**) AWC phenotypes of mosaic animals containing the extrachromosomal transgene *nsy-5p*::*slo-1(T1001Igf); odr-1p*::*DsRed* in only one AWC cell. The data was derived from a subset of data in (**E**). AWC^ON^ was scored as *str-2*-expressing cell; AWC^OFF^ was scored as non-*str-2*-expressing cell. Statistical analysis was performed with a *Z*-test. Error bars represent the standard error of proportion.

To determine the site of *slo-1* and *slo-2* function in promoting AWC^ON^, we performed genetic mosaic analysis in *slo-1(lf); slo-2(lf)* mutants containing an integrated AWC^ON^ marker (*str-2p*::*GFP*) transgene and the extrachromosomal array *odr-3p*::*slo-1(overexpressor (OE))*; *odr-1p*::*DsRed* or *odr-3p*::*slo-2(OE); odr-1p*::*DsRed*. Both *odr-3p*::*slo-1(OE)* and *odr-3p*::*slo-2(OE)* transgenes rescued the 2AWC^OFF^ phenotype in *slo-1(lf); slo-2(lf)* mutants and also caused a slight 2AWC^ON^ overexpression phenotype (Fig 4A and 4C). Since extrachromosomal transgenes are unstable and can be randomly lost at each cell division, the co-injected marker *odr-1p*::*DsRed* (normally expressed in both AWC) was used to indicate the presence of the *slo-1(OE)* or *slo-2(OE)* array in AWC. Specifically, we determined if retention of the *slo-1(OE)* or *slo-2(OE)* array in only a single AWC cell causes a bias of AWC^ON^ choice in that cell when the mosaic animals exhibited a wild-type 1AWC^ON^/1AWC^OFF^ phenotype. We found that the *slo-1(OE); slo-2(lf)* AWC became AWC^ON^ and the *slo-1(lf); slo-2(lf)* AWC became AWC^OFF^ in the majority of mosaic animals in which *slo-1* was expressed only in a single AWC neuron (Fig 4B). Similarly, the *slo-1(lf); slo-2(OE)* AWC became AWC^ON^ and the *slo-1(lf); slo-2(lf)* AWC became AWC^OFF^ when mosaic animals expressed *slo-2* only in a single AWC neuron (Fig 4D). Together, these results support that *slo-1* and *slo-2* act cell autonomously to specify AWC^ON^. We did observe a very small percentage of mosaic animals in which the *slo-1(lf); slo-2(lf)* AWC became AWC^ON^ (Fig 4B and 4D). This suggests that although *slo-1* and *slo-2* have a largely cell-autonomous role in promoting the AWC^ON^ fate, they may also have a nonautonomous role. This is similar to other genes in the AWC asymmetry pathway, such as *nsy-*5 and *nsy-4* which display both autonomous and nonautonomous roles in AWC asymmetry [20,21].

Mosaic analysis was also performed in transgenic lines in which *slo-1(T1001Igf)*, containing the *ky389gf* mutation, was overexpressed in a wild-type background, resulting in a strong 2AWC^ON^ phenotype (Fig 4E). When the transgene was retained in only one of the two AWC cells, the *slo-1(gf)* cell became AWC^ON^ and the wild-type cell became AWC^OFF^ in the majority of mosaic animals (Fig 4F). This result is consistent with a largely cell-autonomous function of *slo-1* in promoting AWC^ON^, and also suggests that the AWC with *slo-1(gf)* activity may become hyperpolarized, allowing the cell to reduce calcium influx and take on the AWC^ON^ subtype.

### SLO-1 and SLO-2 BK potassium channels are localized in the vicinity of UNC-2 and EGL-19 voltage-gated calcium channels in AWC axons

SLO-1 and SLO-2 have overlapping expression patterns and have been suggested to potentially form heteromeric channels [24,28,29]. In addition, it has been shown that BK channels and N-type voltage-gated calcium channels localize in close proximity to achieve functional coupling of these channels [30]. To determine if SLO-1, SLO-2, UNC-2 (N/P/Q-type calcium channels), and EGL-19 (L-type calcium channels) localize in close proximity in AWC, we generated single copy transgenes expressing functional translational reporters driven by the AWC *odr-3* promoter using Mos1-mediated single copy insertion [31–33]. The tagged proteins expressed in these transgenes were functional in rescuing respective mutant phenotypes [34](S1 Fig, Materials and Methods).

**Fig 5:**
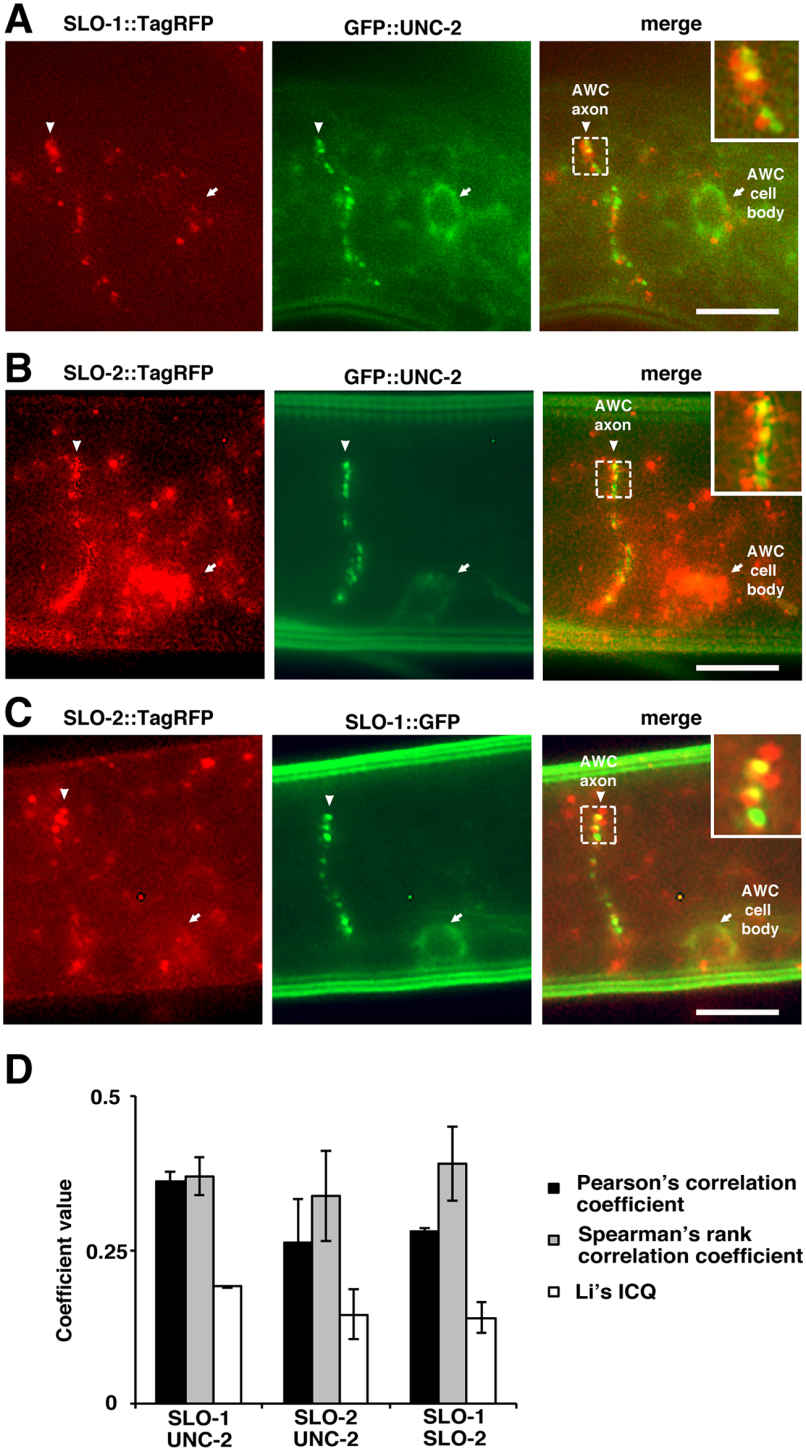
SLO-1 and SLO-2 BK potassium channels are localized in the vicinity of UNC-2 voltage-gated calcium channels in AWC axons. (**A-C**) Images of wild-type L1 animals expressing single copy insertion transgenes *odr-3p*::*slo-1*::*TagRFP* and *odr-3p*::*GFP*::*unc-2* (A), *odr-3p*::*slo-2*::*TagRFP* and *odr-3p*::*GFP*::*unc-2* (B), as well as *odr-3p*::*slo-2*::*TagRFP* and *odr-3p*::*slo-1*::*GFP* (C) in AWC neurons. SLO-1::TagRFP (A), SLO-1::GFP (C), SLO-2::TagRFP (B, C), and GFP::UNC-2 (A, B) were localized in AWC cell bodies (arrows) and in a punctate pattern along AWC axons (arrowheads). In AWC axons, SLO-1::TagRFP was localized next to GFP::UNC-2 (A); SLO-2::TagRFP was adjacent to GFP::UNC-2 (B); and SLO-2::TagRFP was localized near SLO-1::GFP (C). Insets show higher magnification of the outlined areas that exemplify localization of two translational reporters in close proximity. Scale bar, 5 μm. Anterior is at left and ventral is at bottom. (D) Quantification of mean correlation coefficient between SLO-1 and UNC-2, SLO-2 and UNC-2, as well as SLO-1 and SLO-2 using three algorithms of the Coloc 2 plugin in Fiji: Pearson’s correlation coefficient, Spearman’s rank correlation coefficient, and Li’s ICQ. For each colocalization class, images of three animals were used for quantification. Positive values of each coefficient indicate positive correlation, values close to zero indicate no correlation, and negative values indicate anti-correlation. Pearson's correlation coefficient ranges from -1 to +1; Spearman’s rank correlation coefficient ranges from -1 to +1; Li's ICQ value ranges from -0.5 to +0.5. A schematic diagram of the AWC cell body, axon, dendrite, and cilia that represents the approximate region of images in A-C is shown in S2D Fig.

These single copy insertion transgenes showed that GFP::UNC-2, SLO-1::TagRFP, SLO-1::GFP, SLO-2::TagRFP, and GFP::EGL-19 were mainly localized on the plasma membrane of AWC cell bodies and also displayed a punctate pattern along AWC axons (Fig 5 and S2 Fig), similar to the previously shown localization pattern of GFP::UNC-2 in AWC [34]. Since these channels were localized throughout the plasma membrane of the AWC cell body and had distinct punctate patterns in AWC axons, we focused on analyzing their localization in relation to each other in AWC axons. We found that both SLO-1::TagRFP and SLO-2::TagRFP were localized adjacent to GFP::UNC-2 and GFP::EGL-19 in AWC axons (Fig 5A and 5B, S2A and S2B Fig). In addition, SLO-2::TagRFP is located close to SLO-1::GFP in AWC axons (Fig 5C). The Coloc 2 plugin in Fiji was used to quantify colocalization of these proteins in AWC axons using three different algorithms (Pearson’s correlation coefficient, Spearman’s rank correlation coefficient, and Li’s ICQ). Each of the algorithms displayed positive correlation indices (Fig 5D and S2C Fig). This further supports that UNC-2 and EGL-19 localize close to SLO-1 and SLO-2, and that SLO-1 and SLO-2 are localized in close proximity as well.

These results support the notion that BK potassium channels (SLO-1 and SLO-2) and voltage-gated calcium channels (UNC-2 and EGL-19) may function in close proximity for rapid activation of SLO-1 and SLO-2 channels by locally increased calcium levels near UNC-2 and EGL-19 calcium channels.

### *slo-1* and *slo-2* regulate the localization of synaptic markers in AWC neurons

**Fig 6:**
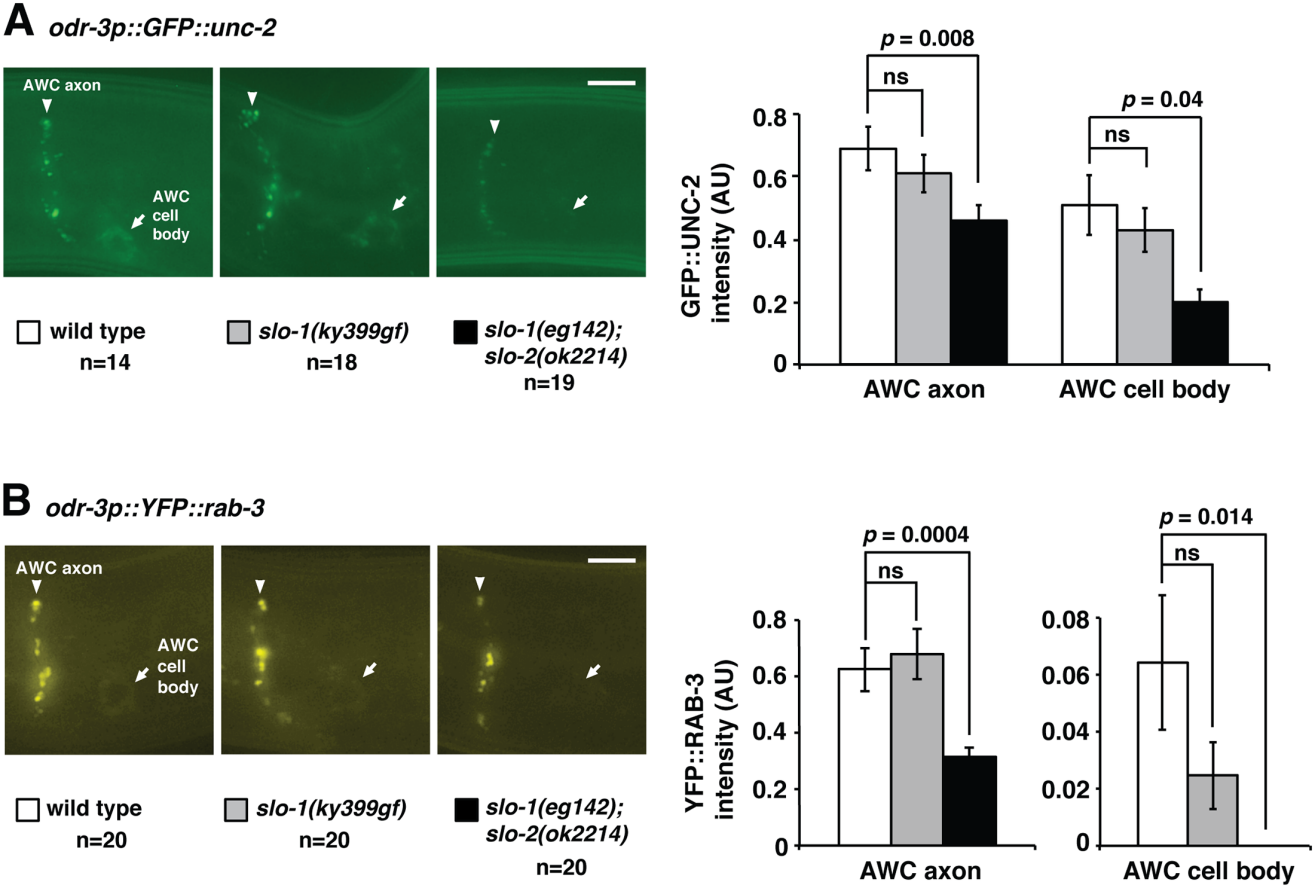
*slo-1* and *slo-2* regulate the subcellular localization of synaptic markers in AWC neurons. **(A**) Left panels: Images of wild type, *slo-1(ky399gf)*, and *slo-1(eg142lf); slo-1(ok2214lf)* mutants expressing the single copy insertion transgene *odr-3p*::*GFP*::*unc-2* (the same transgene as shown in Fig 5A and 5B) in AWC cell bodies (arrows) and axons (arrowheads) in L1. Right panel: Quantification of GFP::UNC-2 fluorescence intensity in AWC axons and cell bodies. *slo-1(eg142lf); slo-1(ok2214lf)* mutants displayed a significant decrease in GFP::UNC-2 intensity in AWC axons and cell bodies. (**B**) Left panels: Images of wild-type, *slo-1(ky399gf)*, and *slo-1(eg142lf); slo-1(ok2214lf)* mutants expressing the single copy insertion transgene *odr-3p*::*YFP*::*rab-3* in AWC cell bodies (arrows) and axons (arrowheads) in L1. Right panel: Quantification of YFP::RAB-3 fluorescence intensity in AWC axons and cell bodies. *slo-1(eg142lf); slo-1(ok2214lf)* mutants had a significant decrease in YFP::RAB-3 intensity in AWC axons and cell bodies. (**A**, **B**) Anterior is at left and ventral is at bottom. Scale bar, 5 μm. Student’s *t-*test was used for statistical analysis. ns, not significant. Error bars, standard error of the mean. AU, arbitrary unit.

It has been shown that communication between the pair of AWC neurons via chemical synapses in axons is important for induction of the AWC^ON^ subtype [11]. Our genetic data suggests that *slo-1* and *slo-2* are required for the specification of the induced AWC^ON^ subtype. In addition, SLO-1 and SLO-2 displayed distinct punctate localization patterns in AWC axons. Thus, we examined whether *slo-1* and *slo-2* regulate localization of synaptic markers in AWC neurons. To do so, we generated Mos1-mediated single copy insertion transgenes expressing fluorescently tagged synaptic markers, GFP::UNC-2 and YFP::RAB-3, driven by the AWC *odr-3* promoter (Figs 5A, 5B and 6). UNC-2 is localized to presynaptic active zones and RAB-3 is a synaptic vesicle marker [34].

In wild type, GFP::UNC-2 was localized in the AWC axon and cell body, and YFP::RAB-3 was mainly localized in a punctate pattern in the AWC axon as shown previously [34] (Fig 6). In *slo-1(ky399gf)* animals, the intensity of GFP::UNC-2 or YFP::RAB-3 was not significantly affected in the AWC axon and cell body (Fig 6). However, *slo-1(eg142lf); slo-2(ok2214lf)* mutants displayed significant reduction in intensity of GFP::UNC-2 and YFP::RAB-3 in the AWC axon and cell body (Fig 6). These results suggest that *slo-1* and *slo-2* are required for localization and/or stability of synaptic markers, UNC-2 and RAB-3, in AWC neurons, which may contribute to the 2AWC^OFF^ phenotype caused by the *slo-1(eg142lf); slo-2(ok2214lf)* mutations. Our genetic mosaic analysis suggests a minor role of nonautonomous function of *slo-1* and *slo-2* in establishing AWC asymmetry (Fig 4), which is consistent with a possible role of *slo-1* and *slo-2* in regulating synaptic communication of AWC neurons. In addition, autofluorescence of the gut found in wild-type animals was visibly decreased in *slo-1(eg142); slo-2(ok2214lf)* mutants (S3A Fig), suggesting that the SLO channels are required for gut autofluorescence.

As a control, the intensity of GFP expressed from the transgene *odr-3p*::*GFP* was analyzed in wild-type and mutant backgrounds, and no significant effect was observed in *slo-1(ky399gf)* and *slo-1(eg142lf); slo-2(ok2214lf)* mutants (S3B Fig). This result rules out the possibility that the activity of the *odr-3* promoter is regulated by *slo-1* and *slo-2*, and also supports the notion that the effect of *slo-1(lf); slo-2(lf)* mutations on UNC-2 and RAB-3 is mainly at the subcellular localization level. It is also possible that *slo-1(lf); slo-2(lf)* mutations may affect *unc-2* and *rab-3* at post-transcriptional levels, such as translation efficiency, mRNA and/or protein stability.

Previous studies have shown that *slo-1(lf)* or *slo-2(lf)* mutations result in increased neurotransmitter release at the neuromuscular junction in the ventral nerve cord [25,35]. However, a recent study showed that UNC-2 localization is not affected at the presynaptic terminals of neuromuscular junctions in *slo-1(lf)* mutants [36]. Thus, previous findings did not demonstrate a correlation between increased neurotransmitter release and increased localization of UNC-2 or RAB-3 at presynaptic sites of the neuromuscular junction in *slo-1(lf)* or *slo-2(lf)* mutants. To examine whether the localization of UNC-2 and RAB-3 is affected in ventral cord motor neurons in *slo-1(lf); slo-2(lf)* mutants, we quantified the intensity of GFP::UNC-2 and RAB-3::mCherry driven by the *unc-25* promoter, which is expressed in ventral cord motor neurons [34]. We examined the axons located anterior to VD5 and DD3 neurons in wild type and *slo-1(lf); slo-2(lf)* mutants at the L4 stage, but no significant difference was observed (S4 Fig). This suggests that *slo-1* and *slo-2* do not play an apparent role in the localization of these presynaptic markers in the ventral nerve cord. The different effects of *slo-1* and *slo-2* mutations on the localization of synaptic markers in AWC neurons and ventral cord motor neurons suggest that *slo-1* and *slo-2* take on a different function in AWC neurons than in the ventral cord motor neurons.

Although no apparent effect of *slo-1(lf); slo-2(lf)* mutations on the localization of UNC-2 and RAB-3 was observed in ventral cord motor neurons, the effect of *slo-1* and *slo-2* mutations on locomotion was performed by analyzing the wavelength and wave width of body wave tracks of wild type, *slo-1(lf)*, *slo-2(lf)*, and *slo-1(lf); slo-2(lf)* animals. We found that the wavelength of the worm track was not affected in the mutants, however the wave width was significantly increased in *slo-1(lf)*, *slo-2(lf)*, and *slo-1(lf); slo-2(lf)* mutants (S5 Fig). These results suggest that *slo-1* and *slo-2* are required for normal locomotion.

### *bkip-1* is required for *slo-1* and *slo-2* function in promoting AWC^ON^

Previous studies have identified several modulators of SLO-1 activity in muscles using forward genetic screens. Since genes may interact in similar pathways in different tissues, we chose these candidate genes to determine whether they may also modulate SLO-1 activity in AWC neurons. *bkip-1* mutants were identified from a screen for suppressors of the lethargic phenotype of *slo-1(gf)* mutants. BKIP-1 (BK channel Interacting Protein), a single pass membrane protein, functions as an auxiliary subunit of SLO-1 to assist in regulating neurotransmitter release and regulate the surface expression of the channel [37]. Similar to *bkip-1*, *ctn-1* (α-catulin), identified from two independent screens for suppressors of the *slo-1(gf)* lethargic phenotype, also regulates surface localization of SLO-1 in both muscles and ventral nerve cord motor neurons [38,39]. In addition, components of the dystrophin-associated protein complex (DAPC), including *dys-1* (dystrophin), *dyb-1* (dystrobrevin), *stn-1* (syntrophin), and *dyc-1* (C-terminal PDZ-domain ligand of nNOS), control the localization of SLO-1 in muscles but not in neurons [40,41]. Furthermore, *islo-1*, encoding a transmembrane protein, functions as an adaptor protein that links the DAPC to SLO-1 for SLO-1 localization in muscles [40].

**Fig 7:**
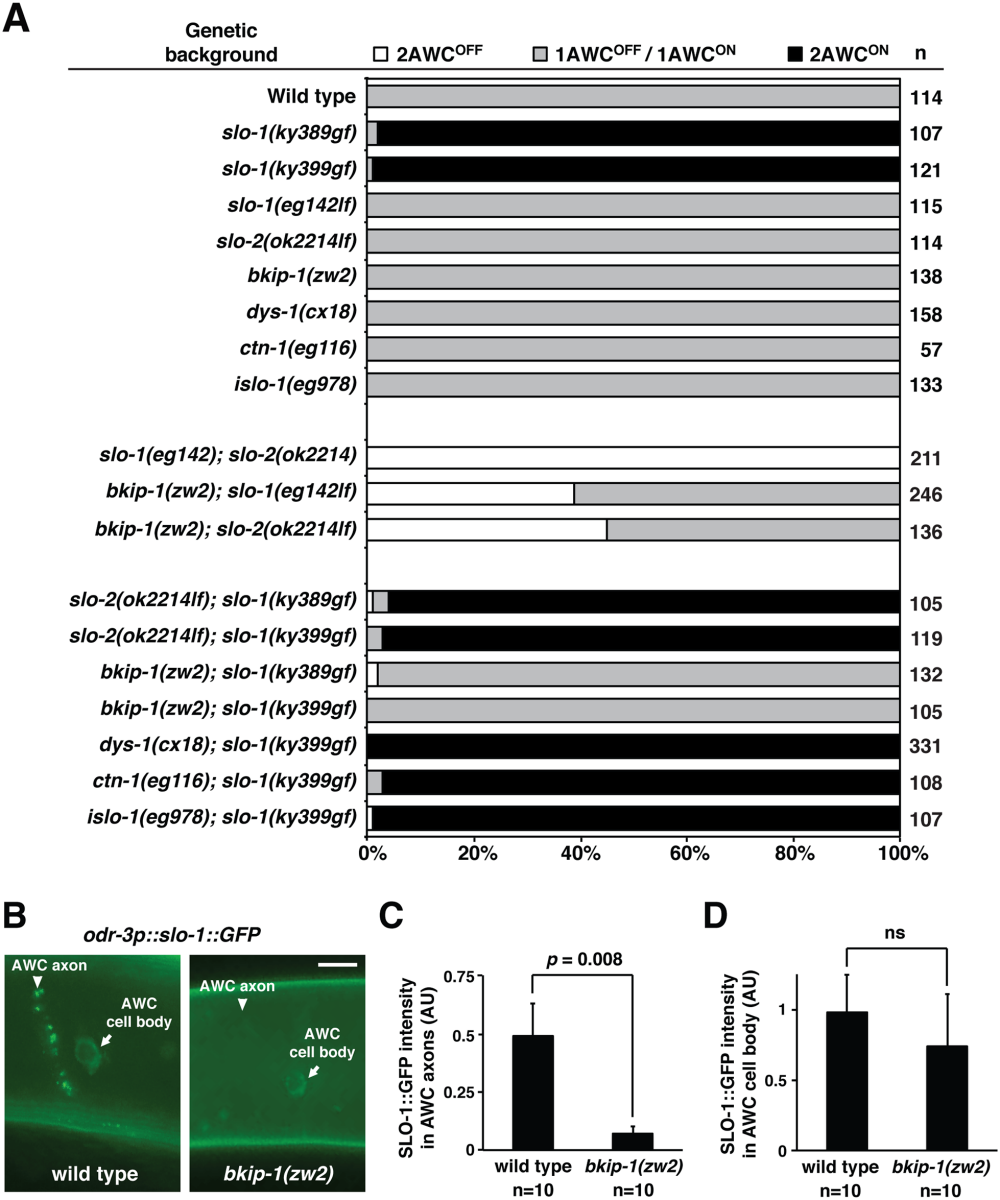
*bkip-1* modulates *slo-1* and *slo-2* activity in AWC neurons. (**A**) Genetic analysis of known modulators of SLO-1 in AWC asymmetry. (**B**) Images of wild type and *bkip-1(zw2)* L1 animals expressing *odr-3p*::*slo-1*::*GFP* in AWC axons and cell bodies. Scale bar, 5 μm. (**C, D**) Quantification of SLO-1::GFP fluorescence intensity in AWC axons (C) and AWC cell body (D). In *bkip-1(zw2)* mutants, SLO-1::GFP intensity is significantly decreased in AWC axons, but is not significantly affected in AWC cell body. Anterior is at left and ventral is at bottom. Student’s *t-*test was used for statistical analysis. ns, not significant. Error bars, standard error of the mean. AU, arbitrary unit.

To determine whether *bkip-1*, *ctn-1*, *dys-1*, and *islo-1* play a role in AWC asymmetry, we first examined expression of the AWC^ON^ marker *str-2p*::*GFP* in their respective loss-of-function mutants, but did not see any defects in AWC asymmetry (Fig 7A). We then determined whether *bkip-1(lf)*, *ctn-1(lf)*, *dys-1(lf)*, and *islo-1(lf)* mutants suppress the *slo-1(gf)* 2AWC^ON^ phenotype in AWC asymmetry by performing double mutant analysis. We found that *dys-1(cx18); slo-1(ky399gf)*, *ctn-1(eg116); slo-1(ky399gf)*, and *islo-1(eg978); slo-1(ky399gf)* all displayed the same 2AWC^ON^ phenotype as *slo-1(ky399gf)* animals (Fig 7A). This suggests that *dys-1*, *ctn-1*, and *islo-1* are not required for *slo-1* function in AWC asymmetry. However, *bkip-1(zw2)* completely suppressed the 2AWC^ON^ phenotype of both *slo-1(ky389gf)* and *slo-1(ky399gf)* mutants to wild type (Fig 7A), indicating that *bkip-1* is required for *slo-1* function in promoting AWC^ON^. As shown by our results, *slo-1(lf)* and *slo-2(lf)* single mutants did not display AWC asymmetry defects (Figs 1B and 7A). However, both *bkip-1(lf); slo-1(lf)* and *bkip-1(lf); slo-2(lf)* displayed a 2AWC^OFF^ phenotype (Fig 7A), supporting a role of *bkip-1* in both *slo-2* and *slo-1* function, respectively. However, the 2AWC^OFF^ phenotype of *bkip-1(lf)*; *slo-1(lf)* and *bkip-1(lf)*; *slo-2(lf)* was not 100% as seen in *slo-1(lf); slo-2(lf)* double mutants (Fig 7A), suggesting that *bkip-1* is not the only factor required for *slo-1* and *slo-2* function in AWC asymmetry. We also determined whether *slo-2* is required for *slo-1* function by crossing *slo-2(lf)* mutants into both *slo-1(ky389gf)* and *slo-1(ky399gf)* alleles. We found that *slo-2(lf)* did not suppress the *slo-1(gf)* 2AWC^ON^ phenotype (Fig 7A), suggesting that *slo-2* is not required for *slo-1* function in AWC asymmetry.

Previous work demonstrates a role of *bkip-1* in regulating the surface expression of SLO-1 in muscle dense bodies and the nerve ring [37]. We therefore determined whether *bkip-1* affects SLO-1 localization in AWC neurons by examining a functional SLO-1::GFP translational reporter driven by the AWC *odr-3* promoter in wild type and *bkip-1(zw2)* mutants (Fig 7B). We found that in *bkip-1(zw2)* mutants, SLO-1::GFP intensity was significantly reduced in AWC axons (Fig 7B and 7C) but is not significantly affected in cell bodies (Fig 7D). This suggests that *bkip-1* is required for appropriate localization of SLO-1 in AWC axons but not in the AWC cell body. Consistent with the result suggesting that *bkip-1* is not the only factor required for *slo-1* function in AWC asymmetry (Fig 7A), this result also suggests that *slo-1* activity could be required in both the AWC axons (dependent on *bkip-1*) and cell bodies (independent of *bkip-1*). We also examined whether *bkip-1(zw2)* mutants display altered the localization of SLO-2::GFP in AWC axons, but did not find a significant effect (S6 Fig). This result suggests that *slo-2* may require *bkip-1* in a manner independent of appropriate localization. *bkip-1* may be required for appropriate *slo-2* expression levels, or BKIP-1 may physically interact with SLO-2.

Together, our results showed that *bkip-1* is the only one of the known modulators of *slo-1* activity in muscles to be also required for *slo-1* and *slo-2* function in AWC asymmetry. Thus, our results suggest that *slo-1* and *slo-2* need a different set of regulators for their function in AWC asymmetry.

### Additional voltage-gated potassium channel EGL-2 (EAG) plays a role in AWC asymmetry

The voltage-dependent activation of SLO-1 and SLO-2 channels is modulated by calcium (for SLO-1 and SLO-2) and chloride (for SLO-2) [24,26]. To determine whether any chloride channels or other voltage-gated potassium channels might be involved in establishing left-right AWC asymmetry, we examined AWC asymmetry in mutants of selective channels that have been shown to be expressed in the nervous system (WormBase). Although the majority of mutants examined did not display an AWC asymmetry defect (S7A Fig), a gain of function mutation in *unc-103* (ERG voltage-gated potassium channel) resulted in a slight 2AWC^ON^ phenotype (S7B Fig). In addition, a gain of function mutation in *egl-2* (EAG voltage-gated potassium channel) caused a high penetrance of the 2AWC^ON^ phenotype (S7B Fig), as previously shown [11]. We found that the *egl-2(n693gf)* mutation suppressed the 2AWC^OFF^ phenotype observed in *slo-1(eg142); slo-2(ok2214)* double mutants, *nsy-5(ky634lf)*, *unc-43(n498gf)*, and *tir-1(ky648gf)* single mutants (S7B Fig). This suggests that *egl-2* may function downstream of these genes to promote the AWC^ON^ fate. Alternatively, it is possible that *egl-2* may function at the same level as *slo-1* and *slo-2*; and that the production of 2 AWC^ON^ neurons by *egl-2(gf)* in the *slo-1(eg142); slo-2(ok2214)* mutants is because *egl-2(gf)* is sufficient to cause enough membrane hyperpolarization to induce AWC^ON^ even in the absence of *slo-1* and *slo-2*. Like *slo-1(lf)* and *slo-2(lf)* mutants, loss-of-function mutations in *egl-2* did not cause a significant effect on AWC asymmetry nor did *slo-1(lf); egl-2(lf)* double mutants (S7B Fig), suggesting that *egl-2* may act redundantly with other factor(s) in promoting AWC^ON^.

## Discussion

Here we identify an essential role of SLO BK potassium channels in asymmetric differentiation of one pair of olfactory neurons. Our findings reveal a functional link between gap junctions and SLO channels in inhibition of voltage-gated calcium channels for diversification of olfactory neurons. To the best of our knowledge, stochastic AWC asymmetry is the first system in which SLO channels are implicated in terminal neuron differentiation, stochastic cell fate determination, and left-right patterning.

**Fig 8:**
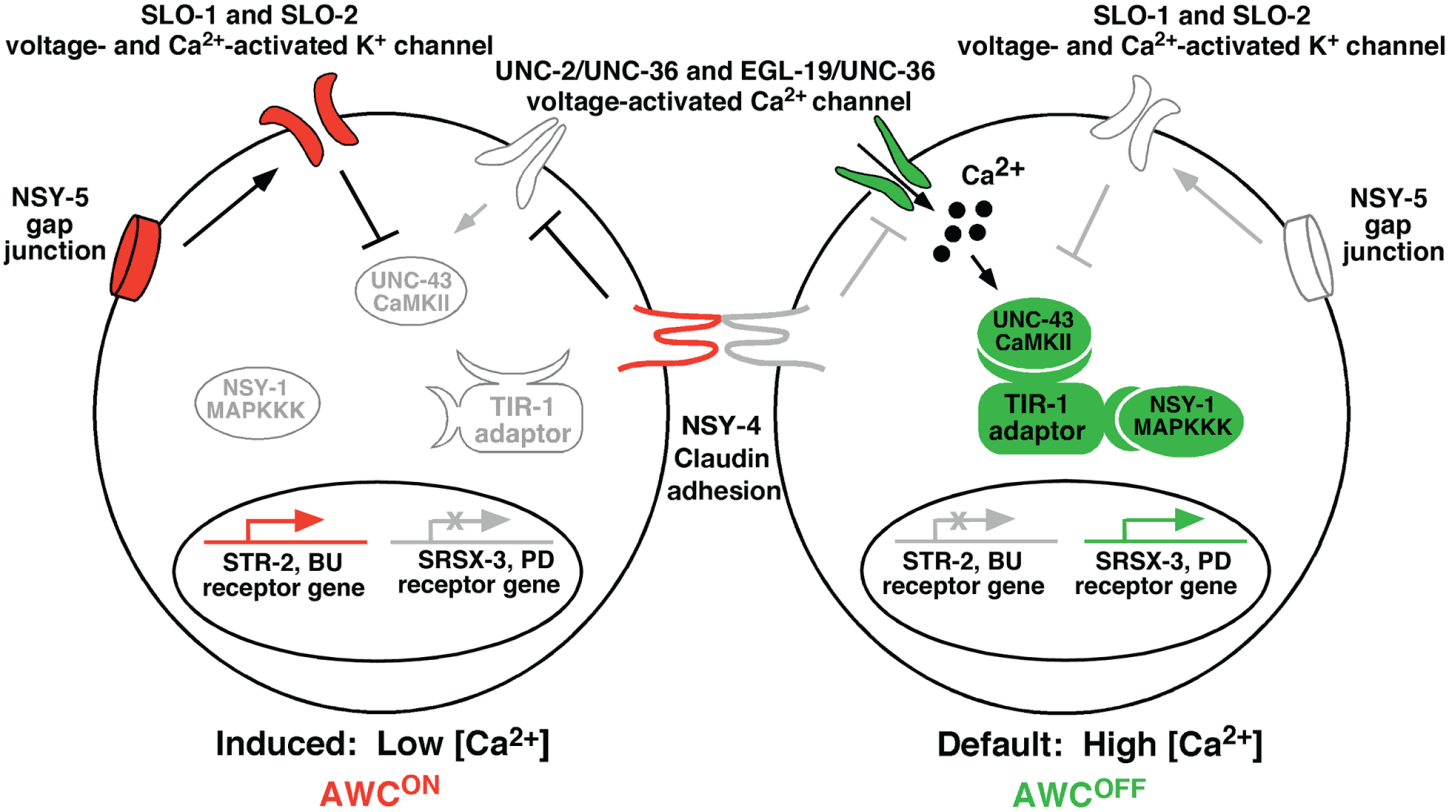
Model of *slo-1* and *slo-2* function in AWC asymmetry. AWC asymmetry is stochastic, and this figure illustrates the case when AWC^ON^ is on the left side of the head. Molecules in green represent AWC^OFF^ promoting, molecules in red represent AWC^ON^ promoting, and those in grey represent less active or inactive molecules. In the AWC^OFF^ neuron (right), calcium enters the cell through voltage-gated calcium channels (UNC-2/UNC-36 and EGL-19/UNC-36) and stimulates a MAP kinase cascade consisting of UNC-43 (CaMKII), TIR-1 (Sarm1) adaptor protein, and NSY-1 (MAPKKK). This leads to expression of the AWC^OFF^ marker *srsx-3* and suppression of the AWC^ON^ marker *str-2*. In the AWC^ON^ cell (left), NSY-5 gap junctions activate SLO-1 and SLO-2 voltage- and calcium-activated potassium channels, which antagonize the function of UNC-2/UNC-36 and EGL-19/UNC-36 calcium channels by suppressing the calcium-activated CaMKII-MAP kinase cascade. NSY-4 (claudin) acts in parallel with NSY-5, SLO-1, and SLO-2 to inhibit calcium channel-mediated signaling, resulting in de-repression of *str-2* expression.

Our results suggest antagonistic and parallel functions of BK potassium channels (SLO-1 and SLO-2) and voltage-gated calcium channels (UNC-2/UNC-36 and EGL-19/UNC-36) downstream of NSY-5 gap junctions in AWC asymmetry. UNC-2/UNC-36 and EGL-19/UNC-36 activate a CaMKII-MAP kinase cascade to specify the default AWC^OFF^ subtype, while SLO-1 and SLO-2 inhibit the calcium channel-activated kinase cascade to promote the induced AWC^ON^ subtype (Fig 8). Calcium and voltage are potential signals that mediate intercellular communication between the two AWC neurons and other neurons in the NSY-5 gap junction network to coordinate stochastic AWC asymmetry [19,20]. In addition, both SLO BK channels and voltage-gated calcium channels generate voltage and calcium signals, and are subject to calcium- and voltage-dependent activation and inactivation [26,42]. The regulatory loop between gap junctions, SLO BK channels, and voltage-gated calcium channels can potentially generate sustained differences in calcium-regulated signaling outputs between the two AWC cells through positive and negative feedback mechanisms, leading to asymmetric differentiation of AWC cells. This extends the previous model of NSY-5 function in AWC asymmetry by identifying SLO BK channels as the mediators of transient gap junction signaling for antagonizing voltage-gated calcium channel pathways.

Signaling via NSY-5 gap junctions may lead to transcriptional regulation of *slo-1* and *slo-2* in order to ensure that these genes are expressed asymmetrically in the AWC neurons. Studies have shown that connexin gap junction proteins are capable of regulating gene expression. For example, gap junction communication mediated by Cx43 is required for ERK phosphorylation of the transcription factor Sp1, which in turn leads to appropriate expression of an osteoclastin transcriptional element [43]. It has been suggested that gap junctions may allow diffusion of second messengers such as calcium and cyclic nucleotides, which subsequently can influence gene transcription [43,44]. It has also been suggested that C-terminal tails of connexins may bind to particular proteins, which can then contribute to regulating gene expression [44]. It is possible that NSY-5 gap junctions use similar mechanisms to regulate *slo-1* and *slo-2* gene expression.

SLO-1 is 55% identical to its mouse orthologue Slo1 and SLO-2 is 41% identical to its mammalian orthologue Slack, while SLO-1 is only 18% identical to its nematode paralogue SLO-2 along the entire channel peptide [28]. SLO-1/Slo1 and SLO-2/Slack have overlapping expression patterns and may form heteromeric channels [24,28,29]. However, functional relationships between SLO-1/Slo1 and SLO-2/Slack have not yet been demonstrated in any biological contexts. Our results show that SLO-1 localizes in close proximity to SLO-2 in AWC neurons. In addition, our results suggest that *slo-1* and *slo-2* have complete functional redundancy in AWC asymmetry, since loss-of-function mutations in either gene alone did not cause any defects in AWC asymmetry while *slo-1(lf); slo-2(lf)* double mutants displayed a complete penetrance of the 2AWC^OFF^ phenotype. Functional redundancy between SLO-1/Slo1 and SLO-2/Slack may represent one of the general mechanisms for their roles in other systems.

The voltage range of activation of BK channels is modulated by different intracellular factors including calcium (for SLO-1, Slo1, and SLO-2), chloride (for SLO-2 and Slack), sodium (for Slack), pH, and phosphorylation [24,26]. None of the mutants of chloride channels we examined displayed any AWC asymmetry defects. In addition, although SLO-2 shares a complete redundant function with SLO-1 in AWC asymmetry, it has not been shown that the activation of SLO-1 channels is sensitive to chloride. These findings suggest that SLO-2’s redundant role with SLO-1 in establishing AWC asymmetry may be more dependent on sensitivity to calcium than to chloride.

Calcium-activated BK channels and voltage-gated calcium channels have been shown to localize in close proximity to ensure selective and rapid activation of BK channels by a local increase in cytosolic calcium level [30]. The sensitivity of vertebrate Slo1 channels to calcium provides an important negative feedback for calcium entry in many cell types. For example, activation of Slo1 channels causes transient membrane hyperpolarization, which limits calcium entry through voltage-gated calcium channels to control the burst of calcium action potentials in cerebellar Purkinje cells and to regulate synaptic transmission in presynaptic terminals [26]. Our genetic results and findings that SLO-1 and SLO-2 localize close to UNC-2 and EGL-19 voltage-gated calcium channels are consistent with the physiological roles of vertebrate Slo1 channels in inhibiting voltage-gated calcium channels through functional coupling and negative feedback. By analogy to functional coupling between Slo1 and voltage-gated calcium channels in vertebrates, SLO-1 and SLO-2 may couple with UNC-2/UNC-36 and EGL-19/UNC-36 to generate oscillation of cytosolic calcium and voltage signals to coordinate stochastic AWC asymmetry through a feedback loop. In this hypothetical feedback loop, an increase in voltage triggers voltage-gated calcium channels to open, leading to an increase in intracellular calcium levels. High calcium levels allow the coupled calcium-activated BK channels to open, resulting in a decrease in voltage. The decreased voltage causes the voltage-gated calcium channels to close, leading to a decrease in intracellular free calcium levels and the subsequent closure of calcium-activated BK channels and an increase in voltage. This would initiate another cycle of calcium and voltage oscillation. Previous studies identified two forms of intercellular communication important for AWC asymmetry: one is mediated by NSY-5 gap junctions between the cell body of AWC and other neurons in a network [19,20]; the other is by synaptic connection between two AWC axons [10,11]. Since SLO-1 and SLO-2 are localized in proximity to UNC-2 and EGL-19 at the AWC axons, functional coupling between BK channels (SLO-1 and SLO-2) and voltage-activated calcium channels (UNC-2 and EGL-19) may occur at AWC axons.

Our study has revealed that SLO-1 and SLO-2 have different functions and interacting partners in AWC olfactory neurons than in ventral cord motor neurons. Our genetic analysis suggests that BK potassium channels (SLO-1 and SLO-2) act to antagonize calcium channels (UNC-2/UNC-36 and EGL-19/UNC-36) to promote the AWC^ON^ identity. A recent report suggests that in M4 motor neurons, UNC-2 and UNC-36 function to activate SLO-1, which in turn antagonizes the EGL-19 calcium channel to inhibit synaptic transmission at the M4 neuromuscular junction [45]. A recent study showed that UNC-2 localization is not affected at the presynaptic terminals of neuromuscular junctions in *slo-1(lf)* mutants [36]. However, our results suggest that *slo-1* and *slo-2* are required for appropriate localization or stability of presynaptic markers UNC-2 and RAB-3 in AWC axons. We also show that SLO-1 and SLO-2 localize in close proximity to both UNC-2 and EGL-19 calcium channels in AWC neurons, in contrast to a report that SLO-2 exclusively couples with EGL-19 but not with UNC-2 in ventral cord motor neurons [35].

BK channels are ubiquitously expressed and have a staggering repertoire of functions in different tissues. To achieve functional diversity, BK channels, which assemble as tetramers of pore-forming α-subunits, can form complexes with various auxiliary β-subunits. For example, the β1 subunit changes gating and calcium sensitivity of Slo1 α subunits, and β2 subunits promote fast inactivation of Slo1 channels [26]. In addition, functional diversity of Slo1 channels can be achieved by alternative splicing, posttranslational modifications, and heteromultimer formation [26]. In *C*. *elegans*, several modulators have been identified for surface expression and activity of SLO-1 channels in muscles and neurons [37–41]. Our results show that the auxiliary subunit BKIP-1 is the only previously identified modulator of SLO-1 to be required for SLO-1 and SLO-2 function in asymmetric AWC differentiation. AWC asymmetry may provide an effective model system to identify novel modulators of SLO BK channels *in vivo* due to the ease of unbiased forward genetic screens in identifying biologically relevant genes and robust phenotypic readouts of SLO channel activity.

## Materials and Methods

### Strains and transgenes

Wild type is strain N2, *C*. *elegans* variety Bristol. Strains were maintained by standard methods [46]. Mutants used were as follows: *nsy-5(ky634)* I [20], *dys-1(cx18)* I [47], *ctn-1(eg116)* I [38], *avr-14(ad1302)* I [48], *bkip-1(zw2)* II [37], *clh-1(qa900)* II, *clh-1(qa901)* II, *clh-2(ok636)* II, *clh-3(ok763)* II, *unc-36(e251)* III [46], *tir-1(ky648gf)* III [18], *nsy-4(ky627)* IV [21], *unc-103(e1597gf)* III, *egl-19(n582)* IV [49], *islo-1(eg978)* IV [40], *unc-43(n498gf)* IV [50], *unc-43(n1186)* IV, *slo-1(ky399gf)* V [11], *slo-1(ky389gf)* V [11], *slo-1(eg142)* V [40], *slo-1(js118)* V [25], *slo-1(js379)* V [25], *egl-2(n693gf)* V, *egl-2(n693n904)* V, *exp-2(sa26ad1426)* V, *shw-3(ok1884)* V, *clh-6(ok791)* V, *avr-15(ad1051)* V, *slo-2(ok2214)* X (*C*. *elegans* knockout consortium), *slo-2(nf100)* X [51], *egl-36(n728)* X, *egl-36(n728n398)* X, and *clh-4(ok1162)* X, *unc-2(lj1)* X [52].

Integrated transgenes used include *kyIs140* [*str-2p*::*GFP; lin-15(+)*] I [11], *vyIs76* [*ceh-36p*::*myrTagRFP; ofm-1p*::*DsRed*] I, *vyIs58* [*odr-1p*::*TagRFP*] I, *vyIs56* [*odr-1p*::*TagRFP*] III, *vyIs68* [*str-2p*::*TagRFP*; *srsx-3p*::*GFP*] II [7], *vySi8* [*odr-3p*::*slo-2c*::*TagRFP; unc-119(+)*] II, *vySi18* [*odr-3p*::*GFP*::*unc-2; unc-119(+)*] II, *vySi23* [*odr-3p*::*slo-1a*::*TagRFP; unc-119(+)*] II, *vySi38* [*odr-3p*::*slo-1a*::*GFP; unc-119(+)*] II, *vySi39* [*odr-3p*::*YFP*::*rab-3; unc-119(+)*] II, *vySi58* [*odr-3p*::*slo-2*::*TagRFP; unc-119(+)*] IV, *vyIs51* [*str-2p*::*2xnlsTagRFP; ofm-1p*::*DsRed*] V [18], *vyIs74* [*ceh-36p*::*myrTagRFP; ofm-1p*::*DsRed*] V, *otIs264* [*ceh-36p*::*TagRFP*] [53], *kyIs479* [*unc-25p*::*GFP*::*unc-2; unc-25*::*mCherry*::*rab-3; odr-1p*::*mCherry*] [34], *vyTi2* [*odr-3p*::*GFP*::*egl-19*], and *vyTi4* [*odr-3p*::*GFP*::*egl-19*]. Transgenes maintained as extrachromosomal arrays include *vyEx842*, *843* [*nsy-5p*::*slo-1* (7.5 ng/μl); *odr-1p*::*DsRed* (15 ng/μl); *ofm-1p*::*DsRed* (30 ng/μl)], *vyEx1573*, *1574*, *1575*, *1576* [*nsy-5p*::*slo-1(T1001Igf)* (7.5 ng/μl); *odr-1p*::*DsRed* (15 ng/μl); *ofm-1p*::*DsRed* (30 ng/μl)], *vyEx822*, *823* [*nsy-5p*::*slo-1(E350Kgf)* (7.5 ng/μl); *odr-1p*::*DsRed* (15 ng/μl); *ofm-1p*::*DsRed* (30 ng/μl)], *vyEx1539*, *1540* [*slo-1p*::*GFP* (15 ng/μl); *ofm-1*::*DsRed* (30 ng/μl)], *vyEx1684* [*slo-1p*::*2xnlsGFP* (5 ng/μl)], *vyEx1701* [*slo-1p*::*2xnlsGFP* (2 ng/μl; *pRF4(rol-6(su1006)* (50 ng/μl)], *sEx10749* [*slo-2p*::*GFP; pCeh361*], *vyEx1122*, *1151* [*odr-3p*::*slo-1* (30 ng/μl); *odr-1p*::*DsRed* (15 ng/μl); *ofm-1p*::*DsRed* (30 ng/μl)], *vyEx1682* [*ceh-36p*::*myrTagRFP* (5 ng/μl)], *vyEx1239* [*odr-3p*::*slo-2c* (30 ng/μl); *odr-1p*::*DsRed* (15 ng/μl); *ofm-1p*::*DsRed* (30 ng/μl)], *vyEx1572* [*odr-3p*::*slo-2d* (30 ng/μl); *odr-1p*::*DsRed* (15 ng/μl); *ofm-1p*::*DsRed* (30 ng/μl)], *vyEx1418* [*odr-3p*::*slo-1*::*GFP* (20 ng/μl); *ofm-1p*::*DsRed* (30 ng/μl], *vyEx1393*, *1367* [*slo-1p*::*slo-1* (15 ng/μl); *odr-1p*::*DsRed* (15 ng/μl); *ofm-1p*::*DsRed* (30 ng/μl)], *vyEx1266* [*slo-1p*::*slo-1*::*GFP* (7.5 ng/μl); *ofm-1p*::*DsRed* (30 ng/μl)], *vyEx1594* [*odr-3p*::*slo-1*::*TagRFP* (30 ng/μl); *ofm-1p*::*DsRed* (30 ng/μl)], *vyEx1325*, *1326* [*odr-3p*::*slo-2c*::*TagRFP* (30 ng/μl); *elt-2p*::*GFP* (5 ngl/μl)], and *vyEx611* [*odr-3p*::*GFP* (7.5 ng/μl); *elt-2p*::*CFP* (7.5 ng/μl)].

### Plasmid construction

To make *nsy-5p*::*slo-1*, a 3420 bp fragment of full-length *slo-1a* cDNA was amplified from *snb-1p*::*slo-1a* (pBK3-1) [25] and cloned into a vector containing a 5556 bp of *nsy-5* promoter [20]. *nsy-5p*::*slo-1(T1001Igf) and nsy-5p*::*slo-1(E350Kgf)* were generated by site directed mutagenesis of *nsy-5p*::*slo-1* using a QuikChange II XL Site-Directed Mutagenesis Kit (Stratagene). *slo-1p*::*GFP* was made by replacing *slo-1a*::*GFP* in the *slo-1p*::*slo-1a*::*GFP* vector containing a 5239 bp of *slo-1* promoter [25] with GFP. *slo-1p*::*slo-1* was generated by subcloning the 3420 bp of *slo-1a* coding region into the vector containing a 5239 bp of *slo-1* promoter. *odr-3p*::*slo-1* was made by subcloning the 3420 bp of *slo-1a* coding region into a vector containing the *odr-3* promoter (Roayaie et al. 1998). *odr-3p*::*slo-2c* and *odr-3p*::*slo-2d* were generated by cloning 3261 bp of *slo-2c* and 3351 bp of *slo-2d*, respectively, into the *odr-3p* vector.

*odr-3p*::*slo-1*::*GFP* was made by subcloning the *slo-1a*::*GFP* translation fusion from *slo-1p*::*slo-1a*::*GFP* [25] into the *odr-3p* vector. To make *odr-3p*::*slo-1*::*TagRFP*, TagRFP was inserted into the *slo-1a* cDNA at a location corresponding to a region of the protein between S8 and S9, the same insertion site as GFP in *slo-1p*::*slo-1a*::*GFP* [25], using fusion PCR. *odr-3p*::*slo-2c*::*TagRFP* was made by inserting TagRFP into the *slo-2c* cDNA at a location corresponding to a region of the protein between the last 16^th^ and 15^th^ amino acids from the C-terminus, the same insertion site as GFP in a *slo-2*::*GFP* translation fusion construct [28], using fusion PCR. For Mos1-mediated single copy insertion (MosSCI) of these translational reporter transgenes, a pAB1 construct [14], derived from a pCFJ151 MosSCI insertion vector for integration on chromosome II [31], was modified to generate pAB1.1 that includes a new set of restriction enzyme sites. *odr-3p*::*slo-1*::*TagRFP*, *odr-3p*::*slo-2*::*TagRFP*, and *odr-3p*::*YFP*::*rab-3* fragments were subcloned into pAB1.1 to generate *pAB1*.*1*::*odr-3p*::*slo-1*::*TagRFP*, *pAB1*.*1*::*odr-3p*::*slo-2*::*TagRFP*, and *pAB1*.*1*::*odr-3p*::*YFP*::*rab-3* respectively. To make *pAB1*.*1*::*odr-3p*::*GFP*::*unc-2*, partially overlapped fragments of linearized pAB1.1 vector backbone as well as *odr-3p*::*GFP* and *GFP*::*unc-2*, both of which were PCR amplified from *odr-3p*::*GFP*::*unc-2* [34], were assembled and ligated using Gibson Assembly (New England Biolabs; Ipsiwich, MA). *pAB1*.*1*::*odr-3p*::*slo-1*::*GFP* was made by Gibson Assembly of 3 partially overlapped fragments of pAB1.1::*odr-3p* vector backbone linearized from *pAB1*.*1*::*odr-3p*::*slo-2*::*TagRFP*, *odr-3p*::*slo-1*::*GFP*, and *unc-54 3’UTR*. *odr-3p*::*slo-2*::*TagRFP* fragment was cloned into the pCFJ356 MosSCI insertion vector for integration on chromosome IV [33] to generate *pCFJ356*::*odr-3p*::*slo-2*::*TagRFP*. *odr-3p*::*GFP*::*egl-19* miniMos construct was generated by replacing *snt-1p*::*HALO* in the *snt-1p*::*HALO*::*egl-19* miniMos construct (pSAM354), containing a section of *egl-19* gDNA (exons 5–9 and introns in between the exons) sandwiched between two stretches of cDNA (exons 1–4 and 10–17), with an *odr-3p*::*GFP* fragment from *odr-3p*::*GFP*::*unc-2* [33]. We found that a *set-18p*::*GFP*::*egl-19* transgenic array rescued the locomotory phenotypes of the *egl-19(n582)* hypomorph mutant, supporting that GFP::EGL-19 translational reporter is functional.

### Germ line transformation

Transgenic strains were generated by injecting DNA constructs into the syncytial gonad of adult worms (P_0_) as previously described [54]. F_1_ worms expressing fluorescent transgenes were picked and cloned (1 worm per plate). The F_1_ clones that have F_2_ progeny containing fluorescent transgenes were selected as transgenic lines and analyzed.

### MosSCI integrations

MosSCI lines were generated using the direct insertion protocol as previously described [31,33]. Briefly, *pAB1*.*1*::*odr-3p*::*GFP*::*unc-2* (22 ng/μl), *pAB1*.*1*::*odr-3p*::*slo-2*::*TagRFP* (71 ng/μl), *pAB1*.*1*::*odr-3p*::*slo-1*::*TagRFP* (107 ng/μl), *pAB1*.*1*::*odr-3p*::*slo-1*::*GFP* (43 ng/μl), or *pAB1*.*1*::*odr-3p*::*YFP*::*rab-3* (26 ng/μl) was injected along with *hsp16*.*4p*::*peel-1* (10 ng/μl), *eft-3p*::*mos-1* (50 ng/μl), *rab-3p*::*mCherry* (10 ng/μl), *myo-2p*::*mCherry* (2.5 ng/μl), and *myo-3p*::*mCherry* (5 ng/μl) into ~100 EG4322 (*ttTi5605* II; *unc-19(ed3)* III) worms cultured at 15 or 20°C. *pCFJ356*::*odr-3p*::*slo-2*::*TagRFP* (34 ng/μl) was injected along with *hsp16*.*4p*::*peel-1* (10 ng/μl), *eft-3p*::*mos-1* (50 ng/μl), *rab-3p*::*mCherry* (10 ng/μl), *myo-2p*::*mCherry* (2.5 ng/μl), and *myo-3p*::*mCherry* (5 ng/μl) into ~100 EG6703 (*unc-19(ed3)* III; *cxTi10816* IV) worms cultured at 15 or 20°C. Three injected worms were picked to one plate and cultured at 25°C until starvation (~1 week). The starved worms were heat shocked at 34°C for two hours to activate the negative selection marker PEEL-1, which kills animals carrying extrachromosomal arrays. After recovery at 25°C for four hours, worms that were rescued for the *unc-119* phenotype and lacked the three mCherry co-injection markers were cloned out from separate plates. The presence of single copy inserts was verified by PCR.

### miniMOS single copy insertion

miniMos integration was done as previously described [55]. Briefly, *odr-3p*::*GFP*::*egl-19* miniMos construct containing a hygromycin resistance cassette (17.5 ng/μl) was injected along with *hsp16*.*4p*::*peel-1* (10 ng/μl), *eft-3p*::*mos-1* (50 ng/μl), *rab-3p*::*mCherry* (10 ng/μl), *myo-2p*::*mCherry* (2.5 ng/μl), and *myo-3p*::*mCherry* (10 ng/μl) into ~100 *vySi8* II; *unc-119(ed3)* III and *vySi23* II; *unc-119(ed3)* III worms cultured at 15–20°C. Three injected animals were picked per plate and cultured at 25°C. Three days after injection, hygromysin was added directly onto the plate to a final concentration of 0.25 mg/ml and cultured further until starvation (~1 week). The starved worms were heat shocked at 34°C for two hours to kills animals carrying extrachromosomal arrays. After recovery at 25°C for four hours, worms that survived and lacked the three mCherry co-injection markers were cloned out to determine homozygosity.

### Imaging of transgenic worms expressing fluorescent proteins

Transgenic strains expressing fluorescent markers or fluorescently tagged proteins were mounted onto 2% agarose pads and anesthetized with 5mM sodium azide (Sigma) or 7.5mM levamisole (Sigma). Z-stack images were acquired at room temperature (20–22°C) using Zeiss Axio Imager Z1 or M2 microscopes, each of which is equipped with a motorized focus drive, a Zeiss objective EC Plan-Neofluar 40x/1.30 Oil DIC M27, a Piston GFP bandpass filter set (41025, Chroma Technology), a TRITC filter set (41002c, Chroma Technology), and a Zeiss AxioCam CCD digital camera (MRm for Z1 and 506 mono for M2) driven by the Zeiss imaging software (AxioVision for Z1 and ZEN for M2). For comparison of fluorescence intensity, all animals in each set of experiments were subjected to the same exposure time. Fluorescence intensity was measured with AxioVision or ZEN imaging software or NIH ImageJ image processing software. Since the background autofluorescence varies between some genetic backgrounds (S3A Fig), fluorescence intensity of reporter transgenes was subtracted by background fluorescence intensity to obtain corrected fluorescence intensity. Images shown in Figs 1A, 3A, 3C, 3E, 3G, 5A, 5B, 5C, 6A, 6B and 7B, as well as S2A, S2B, S3A, S3B, S4A and S6A Figs were processed with Adobe Photoshop; the same degree of brightness and contrast adjustment was applied to all images in each set of experiments for comparison of fluorescence intensity (Figs 6A, 6B and 7B, as well as S3A, S3B and S6A Figs).

### Genetic mosaic analysis

Genetic mosaic analysis was performed with various unstable extrachromosomal transgenic arrays in either wild type or *slo-1(eg142lf); slo-2(ok2214lf)* mutants. Three different experiments were performed to determine the sites of *slo-1* and *slo-2* function in AWC asymmetry. *odr-3p*::*slo-1* was injected into *slo-1(lf); slo-2(lf)* mutants to determine whether *slo-1* acts cell autonomously or nonautonomously to rescue the 2AWC^OFF^ mutant phenotype. A similar experiment was performed using the *odr-3p*::*slo-2* extrachromosomal array. In the third experiment, *nsy-5p*::*slo-1(T1001Igf)* was injected into wild-type animals. In all three experiments, the *odr-1p*::*DsRed* marker (expressed in AWC and AWB) was included in the injection mix to serve as an indicator for presence or absence of the extrachromosomal transgene in AWC. The AWC^ON^ and AWC^OFF^ neurons were determined using expression of a stable integrated *str-2p*::*GFP* (AWC^ON^ marker) transgene. Transgenic strains were passed for minimum of six generations to allow the transgenes to stabilize before scoring for mosaic animals.

### Colocalization analysis

Colocalization was quantified using the Coloc 2 plugin (http://fiji.sc/Coloc_2) in Fiji [56]. Three different algorithms were used: Pearson’s correlation coefficient, Spearman’s rank correlation coefficient, and Li’s ICQ. For each colocalization class, images of at least three animals were used for quantification. Positive values of each coefficient indicate positive correlation, values close to zero indicate no correlation, and negative values indicate anti-correlation. Pearson's correlation coefficient ranges from -1 to +1; Spearman’s rank correlation coefficient ranges from -1 to +1; Li's ICQ value ranges from -0.5 to +0.5

### Locomotion analysis

Locomotion analysis was performed on L4 animals in wild type, *slo-1(eg142)*, *slo-2(ok2214)*, *and slo-1(eg142); slo2(ok2214)* animals. Single animals of each genotype were placed on a bacterial lawn and allowed to make tracks. The worm tracks as well as individual worms were imaged and analyzed in ImageJ [57]. All animals were placed on the same batch of NGM plates seeded with the same batch of HB101 and were imaged on the same day. Wavelength was measured as the distance between wave peaks, and at least 3 wavelengths were measured and averaged per animal. The wavelength was normalized by the body length of the animal. Wave width was measured as the distance from the peak to the trough of the worm wave. At least 3 wave widths were measured and averaged per animal. The wave width was normalized by the body length of the animal.

## Supporting Information

S1 Fig (Related to Fig 5) Rescue of *slo-1(lf); slo-2(lf)* 2AWC^OFF^ phenotype by different transgenes. 2AWC^ON^, both AWC cells express *str-2*; 1AWC^OFF^/AWC^ON^, only one of the two AWC cells expresses *str-2*; 2AWC^OFF^, neither AWC cell expresses *str-2*.
(TIF)

S2 Fig (Related to Fig 5) EGL-19 localizes in close proximity to SLO-1 and SLO-2. (**A-B**) Images of wild-type L1 animals expressing single copy insertion transgenes *odr-3p*::*slo-1*::*TagRFP* and *odr-3p*::*GFP*::*egl-19* (A) as well as *odr-3p*::*slo-2*::*TagRFP* and *odr-3p*::*GFP*::*egl-19* (B) in AWC neurons. SLO-1::TagRFP (A), SLO-2::TagRFP (B), and GFP::EGL-19 (A, B) were localized in AWC cell bodies (arrows) and in a punctate pattern along AWC axons (arrowheads). In AWC axons, SLO-1::TagRFP was localized next to GFP::EGL-19 (A); SLO-2::TagRFP was adjacent to GFP::EGL-19 (B). Insets show higher magnification of the outlined areas that exemplify localization of two translational reporters in close proximity. Scale bar, 5 μm. Anterior is at left and ventral is at bottom. (**C**) Quantification of mean correlation coefficient between SLO-1 and EGL-19 as well as SLO-2 and EGL-19 using 3 algorithms of the Coloc 2 plugin in Fiji: Pearson’s correlation coefficient, Spearman’s rank correlation coefficient, and Li’s ICQ. Images of four (SLO-1 and EGL-19) or six (SLO-2 and EGL-19) animals were used for quantification. Positive values of each coefficient indicate positive correlation, values close to zero indicate no correlation, and negative values indicate anti-correlation. Pearson's correlation coefficient ranges from -1 to +1; Spearman’s rank correlation coefficient ranges from -1 to +1; Li's ICQ value ranges from -0.5 to +0.5. (**D**) Schematic diagram of the AWC cell body, axon, dendrite, and cilia. The outlined region represents the approximate region of images shown in Fig 5A, 5B and 5C, S2A and S2B Fig.
(TIF)

S3 Fig (Related to Fig 6) *slo-1* and *slo-2* mutants alter gut autofluorescence but have no effect on expression of GFP from an AWC *odr-3* promoter in AWC cells. (**A**) Representative images taken at identical exposure times of wild-type, *slo-1(ky399gf)*, and *slo-1(eg142lf); slo-2(ok2214lf)* animals expressing the single copy insertion *odr-3p*::*GFP*::*unc-2* (partial head images of the same animals were shown in Fig 6A). The gut autofluorescence of the worm is noticeably decreased in *slo-1(eg142lf); slo-2(ok2214lf)* mutants as compared to wild-type and *slo-1(ky399gf)* mutants. Scale bar, 20 μm. (**B**) Left panels: Images of wild type, *slo-1(ky399gf)*, and *slo-1(eg142lf); slo-1(ok2214lf)* mutants expressing *odr-3p*::*GFP* in AWC in L1. Right panel: Quantification of GFP fluorescence intensity in AWC cell bodies. For each animal, GFP intensity was quantified from the single focal plane with the brightest GFP expression in the AWC cell body and subtracted by background fluorescence intensity. Scale bar, 5 μm. Student’s *t-*test was used for statistical analysis. ns, not significant (*p* = 0.6). Error bars, standard error of the mean. AU, arbitrary unit.
(TIF)

S4 Fig (Related to Fig 6) *slo-1* and *slo-2* mutations do not affect localization of UNC-2 and RAB-3 in axons of ventral cord motor neurons. (**A**) Representative L4 images of wild type and *slo-1(eg142); slo-2(ok2214)* expressing *unc-25p*::*GFP*::*unc-2* (left panels) and *unc-25p*::*rab-3*::*mCherry* (right panels). Scale bar, 20 μm Arrows indicate the axon section analyzed in the left graph of panel (B), arrowheads indicate the axon section analyzed in the right graph of panel (B). Anterior is at left and ventral is at bottom. (**B**) Quantification of GFP::UNC-2 and RAB-3::mCherry in axons anterior to the VD5 neuron (left graph) and DD3 neuron (right graph) in wild type and *slo-1(eg142); slo-2(ok2214)* mutants. Student’s *t-*test was used for statistical analysis. ns, not significant. Error bars, standard error of the mean. AU, arbitrary unit.
(TIF)

S5 Fig (Related to Fig 6) *slo-1* and *slo-2* are required for locomotion behavior. (**A**) Quantification of normalized wavelength of wild type, *slo-1(eg142)*, *slo-2(ok2214)*, and *slo-1(eg142); slo-2(ok2214)* mutants. Student’s *t-*test was used for statistical analysis. Error bars, standard error of the mean. ns, not significant. (**B**) Quantification of normalized wave width of wild type, *slo-1(eg142)*, *slo-2(ok2214)*, and *slo-1(eg142); slo-2(ok2214)* mutants. *slo-1(eg142)*, *slo-2(ok2214)*, and *slo-1(eg142); slo-2(ok2214)* have significantly greater wave widths than wild-type animals. Student’s *t-*test was used for statistical analysis. Error bars, standard error of the mean. (**C**) Schematic of body wave worm tracks and indications of wave width (red) and wavelength (green). Scale bar, 0.1 mm. All animals quantified were at the L4 stage.
(TIF)

S6 Fig (Related to Fig 7) The *bkip-1(lf)* mutation does not affect the localization of SLO-2 in AWC. (**A**) Representative images of wild type and *bkip-1(zw2)* L1 animals expressing *odr-3p*::*slo-2*::*TagRFP* in AWC axons and cell bodies. Scale bar, 5 μm. (**B, C**) Quantification of SLO-2::TagRFP fluorescence intensity in AWC axons (**B**) and AWC cell body **(C)**. In *bkip-1(zw2)* mutants, SLO-2::TagRFP intensity is not significantly decreased in AWC axons or AWC cell body. Anterior is at left and ventral is at bottom. Student’s *t-*test was used for statistical analysis. ns, not significant. Error bars, standard error of the mean. AU, arbitrary unit.
(TIF)

S7 Fig Other voltage-gated potassium and chloride channels in AWC asymmetry. (**A**) Analysis on the effect of mutations in additional voltage-gated potassium channels and chloride channels. 2AWC^ON^, both AWC cells express *str-2*; 1AWC^OFF^/AWC^ON^, only one of the two AWC cells expresses *str-2*; 2AWC^OFF^, neither AWC cell expresses *str-2*. **(B**) Analysis on the effect of mutations in *egl-2* (EAG voltage-gated potassium channel) and *unc-103* (ERG voltage-gated potassium channel) on AWC asymmetry.
(TIF)
