# Supplementary figures and images for: SLO BK Potassium Channels Couple Gap Junctions to Inhibition of Calcium Signaling in Olfactory Neuron Diversification
Source: PLoS Genet. 2016 Jan 15;12(1):e1005654. doi: 10.1371/journal.pgen.1005654 (PMC4714817; doi:10.1371/journal.pgen.1005654)

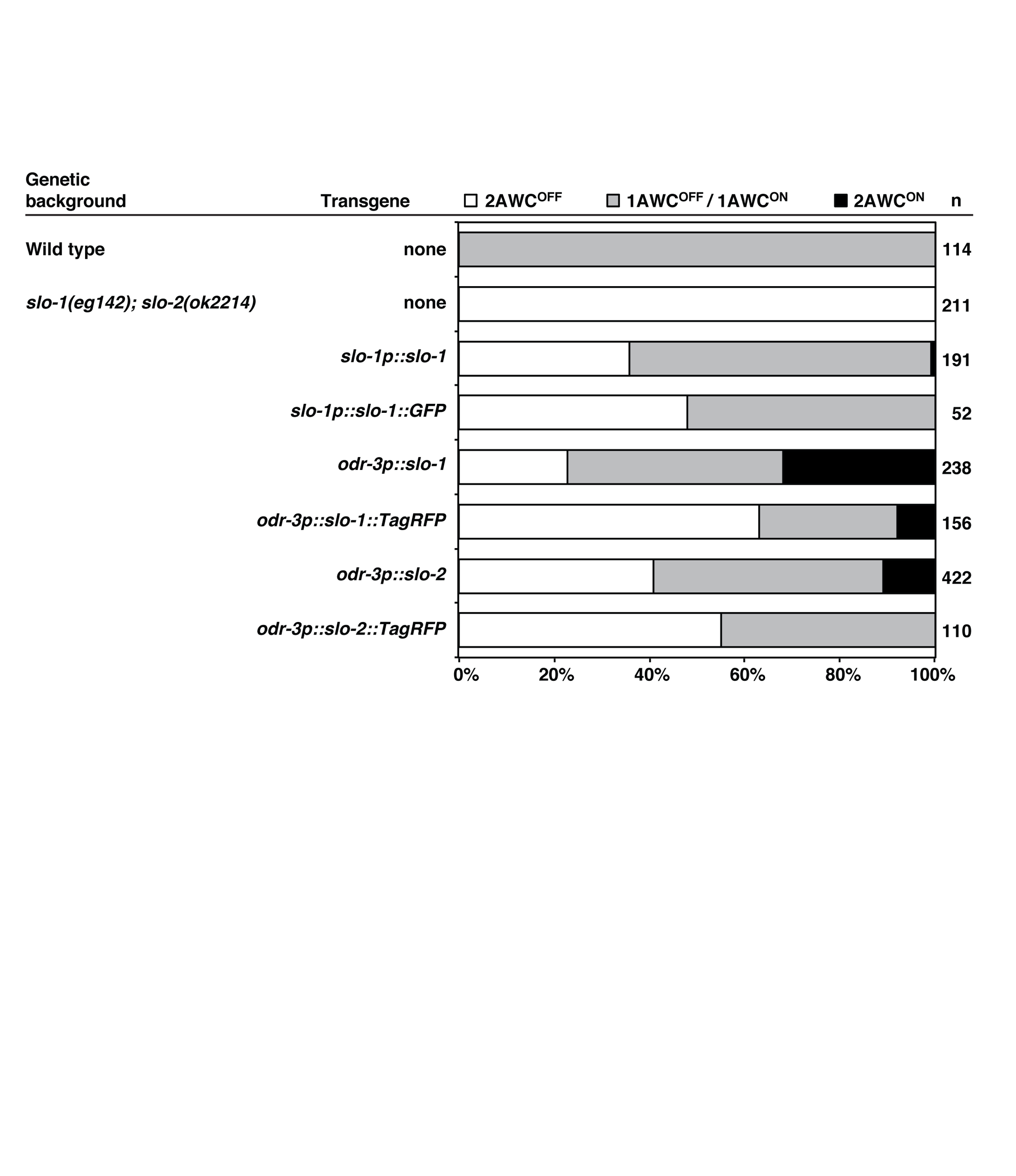

Supplement: S1 Fig — 2AWCON, both AWC cells express str-2; 1AWCOFF/AWCON, only one of the two AWC cells expresses str-2; 2AWCOFF, neither AWC cell expresses str-2. (TIF) [file pgen.1005654.s001.tif]

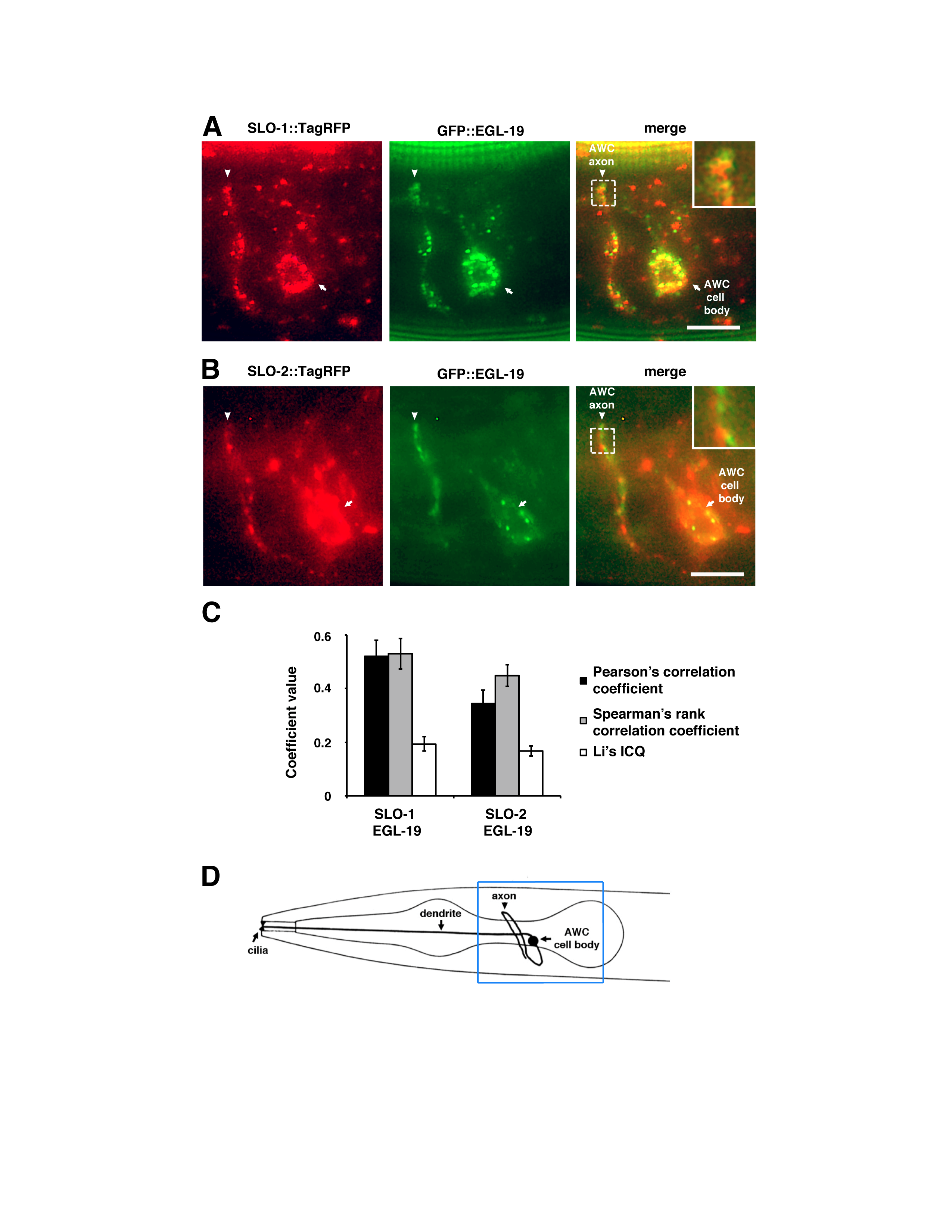

Supplement: S2 Fig — (A-B) Images of wild-type L1 animals expressing single copy insertion transgenes odr-3p::slo-1::TagRFP and odr-3p::GFP::egl-19 (A) as well as odr-3p::slo-2::TagRFP and odr-3p::GFP::egl-19 (B) in AWC neurons. SLO-1::TagRFP (A), SLO-2::TagRFP (B), and GFP::EGL-19 (A, B) were localized in AWC cell bodies (arrows) and in a punctate pattern along AWC axons (arrowheads). In AWC axons, SLO-1::TagRFP was localized next to GFP::EGL-19 (A); SLO-2::TagRFP was adjacent to GFP::EGL-19 (B). Insets show higher magnification of the outlined areas that exemplify localization of two translational reporters in close proximity. Scale bar, 5 μm. Anterior is at left and ventral is at bottom. (C) Quantification of mean correlation coefficient between SLO-1 and EGL-19 as well as SLO-2 and EGL-19 using 3 algorithms of the Coloc 2 plugin in Fiji: Pearson’s correlation coefficient, Spearman’s rank correlation coefficient, and Li’s ICQ. Images of four (SLO-1 and EGL-19) or six (SLO-2 and EGL-19) animals were used for quantification. Positive values of each coefficient indicate positive correlation, values close to zero indicate no correlation, and negative values indicate anti-correlation. Pearson's correlation coefficient ranges from -1 to +1; Spearman’s rank correlation coefficient ranges from -1 to +1; Li's ICQ value ranges from -0.5 to +0.5. (D) Schematic diagram of the AWC cell body, axon, dendrite, and cilia. The outlined region represents the approximate region of images shown in Fig 5A, 5B and 5C, S2A and S2B Fig. (TIF) [file pgen.1005654.s002.tif]

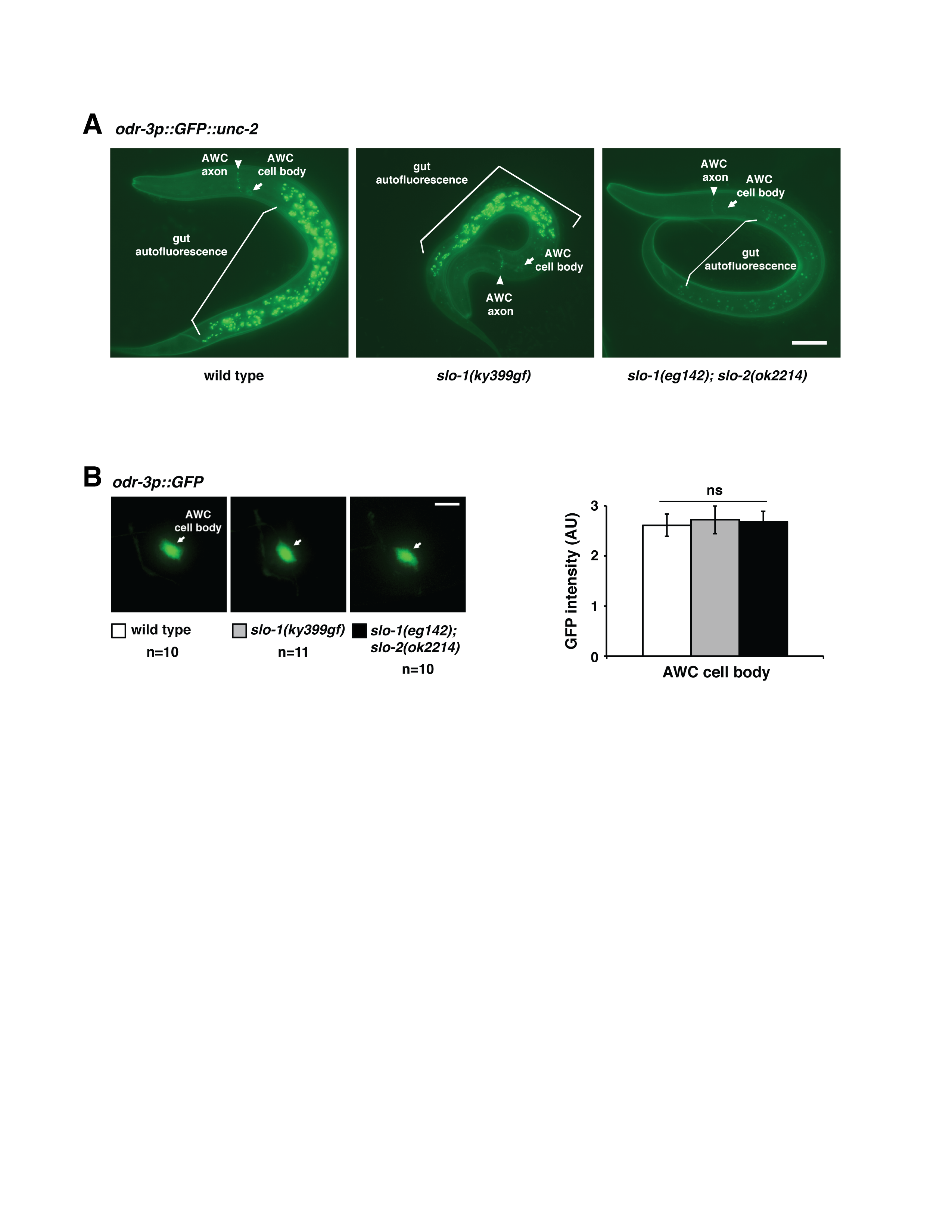

Supplement: S3 Fig — (A) Representative images taken at identical exposure times of wild-type, slo-1(ky399gf), and slo-1(eg142lf); slo-2(ok2214lf) animals expressing the single copy insertion odr-3p::GFP::unc-2 (partial head images of the same animals were shown in Fig 6A). The gut autofluorescence of the worm is noticeably decreased in slo-1(eg142lf); slo-2(ok2214lf) mutants as compared to wild-type and slo-1(ky399gf) mutants. Scale bar, 20 μm. (B) Left panels: Images of wild type, slo-1(ky399gf), and slo-1(eg142lf); slo-1(ok2214lf) mutants expressing odr-3p::GFP in AWC in L1. Right panel: Quantification of GFP fluorescence intensity in AWC cell bodies. For each animal, GFP intensity was quantified from the single focal plane with the brightest GFP expression in the AWC cell body and subtracted by background fluorescence intensity. Scale bar, 5 μm. Student’s t-test was used for statistical analysis. ns, not significant (p = 0.6). Error bars, standard error of the mean. AU, arbitrary unit. (TIF) [file pgen.1005654.s003.tif]

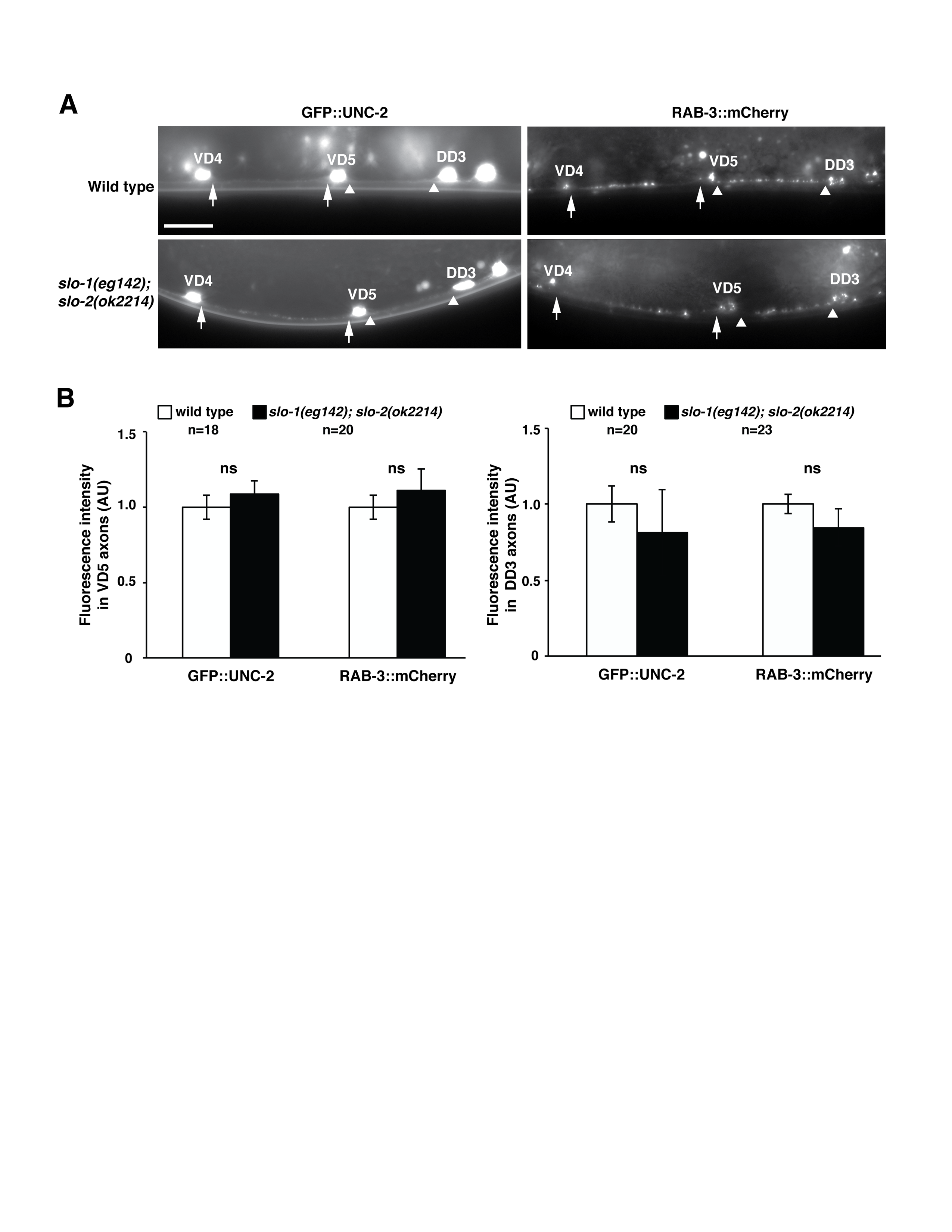

Supplement: S4 Fig — (A) Representative L4 images of wild type and slo-1(eg142); slo-2(ok2214) expressing unc-25p::GFP::unc-2 (left panels) and unc-25p::rab-3::mCherry (right panels). Scale bar, 20 μm Arrows indicate the axon section analyzed in the left graph of panel (B), arrowheads indicate the axon section analyzed in the right graph of panel (B). Anterior is at left and ventral is at bottom. (B) Quantification of GFP::UNC-2 and RAB-3::mCherry in axons anterior to the VD5 neuron (left graph) and DD3 neuron (right graph) in wild type and slo-1(eg142); slo-2(ok2214) mutants. Student’s t-test was used for statistical analysis. ns, not significant. Error bars, standard error of the mean. AU, arbitrary unit. (TIF) [file pgen.1005654.s004.tif]

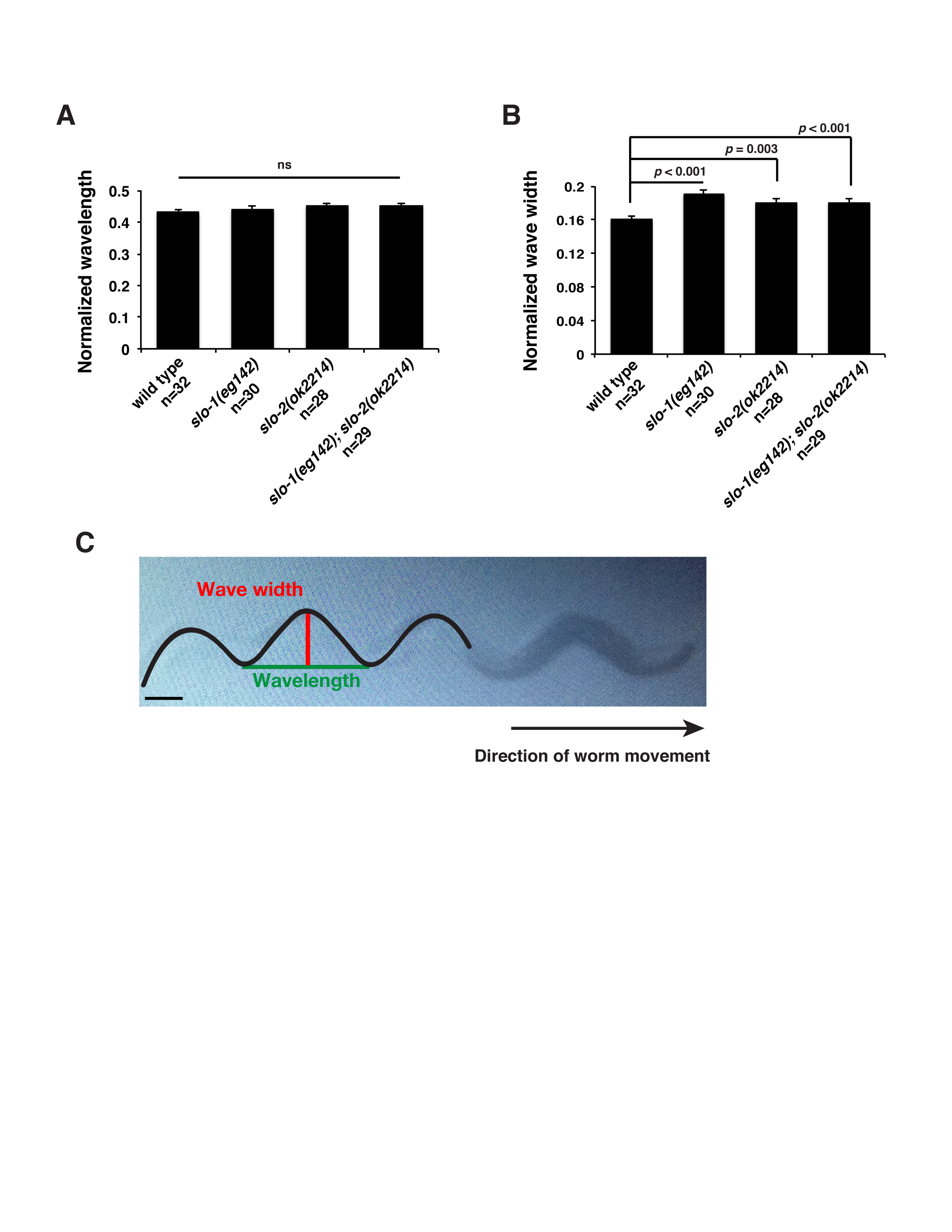

Supplement: S5 Fig — (A) Quantification of normalized wavelength of wild type, slo-1(eg142), slo-2(ok2214), and slo-1(eg142); slo-2(ok2214) mutants. Student’s t-test was used for statistical analysis. Error bars, standard error of the mean. ns, not significant. (B) Quantification of normalized wave width of wild type, slo-1(eg142), slo-2(ok2214), and slo-1(eg142); slo-2(ok2214) mutants. slo-1(eg142), slo-2(ok2214), and slo-1(eg142); slo-2(ok2214) have significantly greater wave widths than wild-type animals. Student’s t-test was used for statistical analysis. Error bars, standard error of the mean. (C) Schematic of body wave worm tracks and indications of wave width (red) and wavelength (green). Scale bar, 0.1 mm. All animals quantified were at the L4 stage. (TIF) [file pgen.1005654.s005.tif]

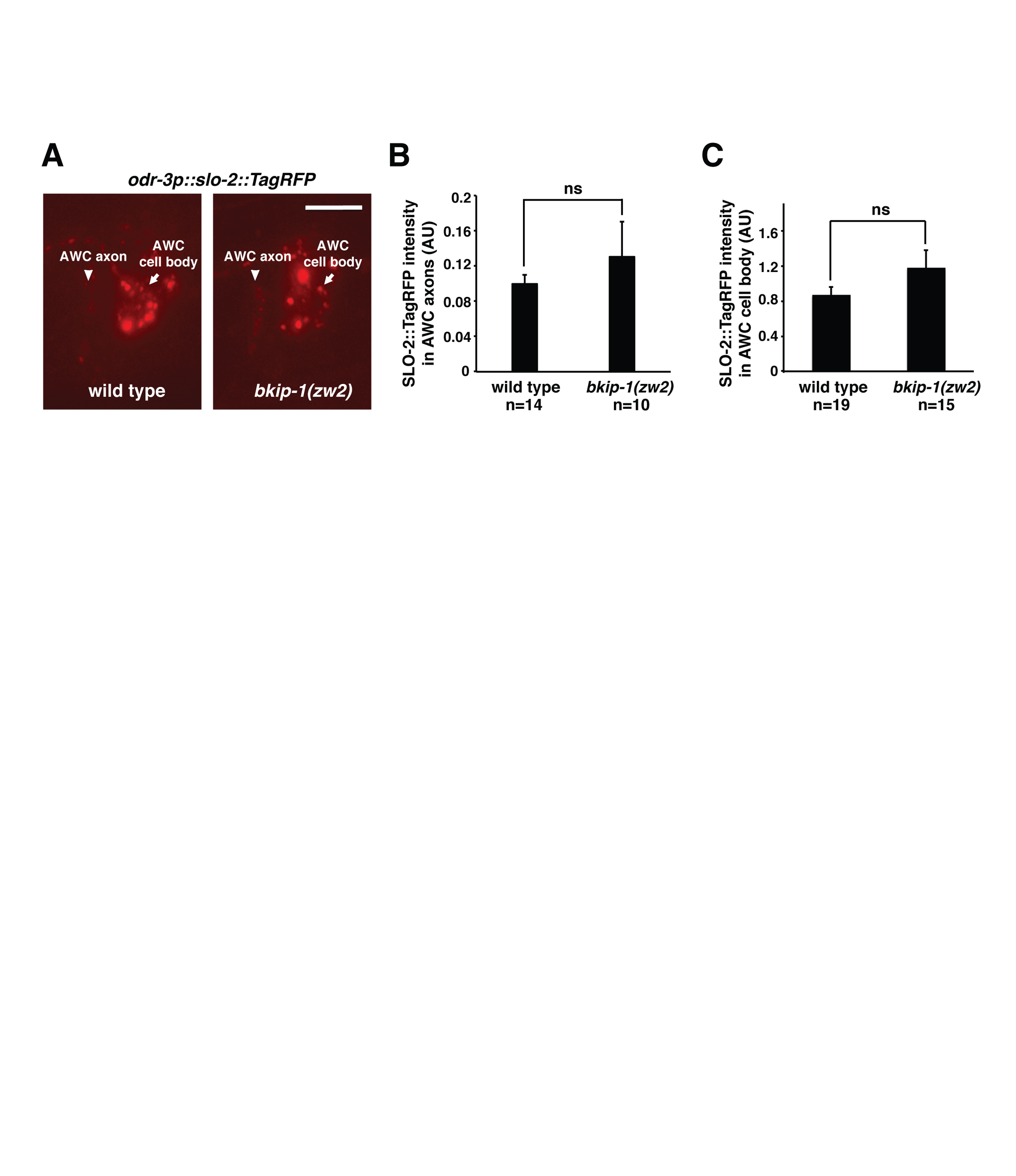

Supplement: S6 Fig — (A) Representative images of wild type and bkip-1(zw2) L1 animals expressing odr-3p::slo-2::TagRFP in AWC axons and cell bodies. Scale bar, 5 μm. (B, C) Quantification of SLO-2::TagRFP fluorescence intensity in AWC axons (B) and AWC cell body (C). In bkip-1(zw2) mutants, SLO-2::TagRFP intensity is not significantly decreased in AWC axons or AWC cell body. Anterior is at left and ventral is at bottom. Student’s t-test was used for statistical analysis. ns, not significant. Error bars, standard error of the mean. AU, arbitrary unit. (TIF) [file pgen.1005654.s006.tif]

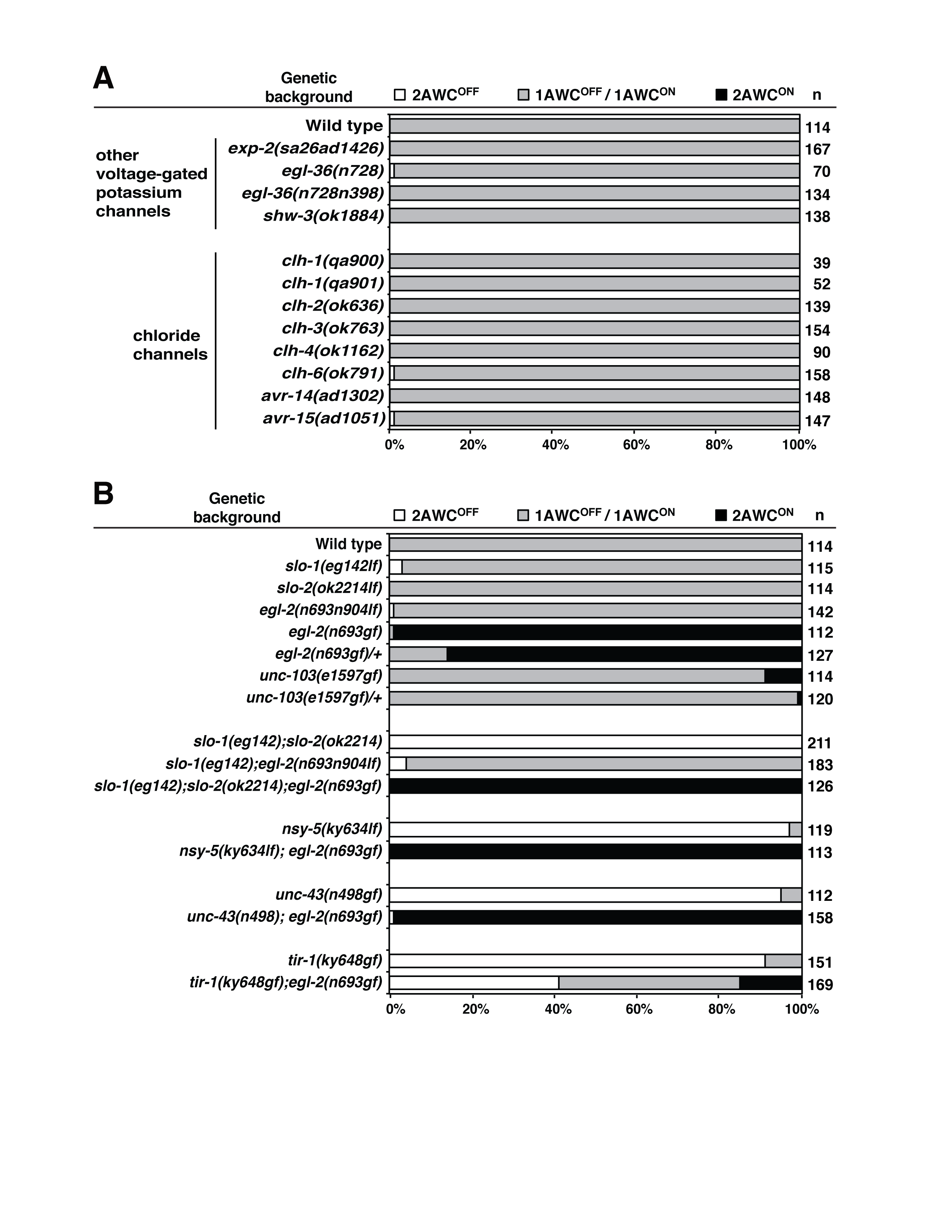

Supplement: S7 Fig — (A) Analysis on the effect of mutations in additional voltage-gated potassium channels and chloride channels. 2AWCON, both AWC cells express str-2; 1AWCOFF/AWCON, only one of the two AWC cells expresses str-2; 2AWCOFF, neither AWC cell expresses str-2. (B) Analysis on the effect of mutations in egl-2 (EAG voltage-gated potassium channel) and unc-103 (ERG voltage-gated potassium channel) on AWC asymmetry. (TIF) [file pgen.1005654.s007.tif]
